# Update on treatments for nonmotor symptoms of Parkinson's disease—an evidence‐based medicine review

**DOI:** 10.1002/mds.27602

**Published:** 2019-01-17

**Authors:** Klaus Seppi, K. Ray Chaudhuri, Miguel Coelho, Susan H. Fox, Regina Katzenschlager, Santiago Perez Lloret, Daniel Weintraub, Cristina Sampaio, and the collaborators of the Parkinson's Disease Update on Non‐Motor Symptoms Study Group on behalf of the Movement Disorders Society Evidence‐Based Medicine Committee, Lana Chahine, Eva‐Maria Hametner, Beatrice Heim, Shen‐Yang Lim, Werner Poewe, Atbin Djamshidian‐Tehrani

**Affiliations:** ^1^ Department of Neurology Medical University Innsbruck Innsbruck Austria; ^2^ Institute of Psychiatry Psychology & Neuroscience at King's College and Parkinson Foundation International Centre of Excellence at King's College Hospital Denmark Hill London United Kingdom; ^3^ Serviço de Neurologia Hospital Santa Maria Instituto de Medicina Molecular Faculdade de Medicina de Lisboa Lisboa Portugal; ^4^ Edmond J Safra Program in Parkinson Disease, Movement Disorder Clinic Toronto Western Hospital, and the University of Toronto Department of Medicine Toronto Ontario Canada; ^5^ Department of Neurology and Karl Landsteiner Institute for Neuroimmunological and Neurodegenerative Disorders Danube Hospital Vienna Austria; ^6^ Institute of Cardiology Research University of Buenos Aires, National Research Council Buenos Aires Argentina; ^7^ Departments of Psychiatry and Neurology Perelman School of Medicine at the University of Pennsylvania Philadelphia Pennsylvania USA; ^8^ Parkinson's Disease and Mental Illness Research Education and Clinical Centers, Philadelphia Veterans Affairs Medical Center Philadelphia Pennsylvania USA; ^9^ CHDI Management/CHDI Foundation Princeton NJ USA; ^10^ Instituto de Medicina Molecular University of Lisbon Lisbon Portugal

**Keywords:** evidence‐based medicine, non‐motor symptoms, Parkinson's disease, randomized controlled trial

## Abstract

**Objective:**

To update evidence‐based medicine recommendations for treating nonmotor symptoms in Parkinson's disease (PD).

**Background:**

The International Parkinson and Movement Disorder Society Evidence‐Based Medicine Committee's recommendations for treatments of PD were first published in 2002, updated in 2011, and now updated again through December 31, 2016.

**Methods:**

Level I studies testing pharmacological, surgical, or nonpharmacological interventions for the treatment of nonmotor symptoms in PD were reviewed. Criteria for inclusion and quality scoring were as previously reported. The disorders covered were a range of neuropsychiatric symptoms, autonomic dysfunction, disorders of sleep and wakefulness, pain, fatigue, impaired olfaction, and ophthalmologic dysfunction. Clinical efficacy, implications for clinical practice, and safety conclusions are reported.

**Results:**

A total of 37 new studies qualified for review. There were no randomized controlled trials that met inclusion criteria for the treatment of anxiety disorders, rapid eye movement sleep behavior disorder, excessive sweating, impaired olfaction, or ophthalmologic dysfunction. We identified clinically useful or possibly useful interventions for the treatment of depression, apathy, impulse control and related disorders, dementia, psychosis, insomnia, daytime sleepiness, drooling, orthostatic hypotension, gastrointestinal dysfunction, urinary dysfunction, erectile dysfunction, fatigue, and pain. There were no clinically useful interventions identified to treat non‐dementia‐level cognitive impairment.

**Conclusions:**

The evidence base for treating a range of nonmotor symptoms in PD has grown substantially in recent years. However, treatment options overall remain limited given the high prevalence and adverse impact of these disorders, so the development and testing of new treatments for nonmotor symptoms in PD remains a top priority. © 2019 The Authors. Movement Disorders published by Wiley Periodicals, Inc. on behalf of International Parkinson and Movement Disorder Society.

The International Parkinson and Movement Disorder Society (MDS) Evidence‐Based Medicine (EBM) Committee regularly publishes recommendations on treating Parkinson's disease (PD) nonmotor symptoms (NMS).^1^, ^2^ An increasing number of studies have been published since the previous review; we review these studies here and present our conclusions.

## Methods

The previous MDS EBM reviews on treatments for NMS of PD reviewed studies from January 2004 to December 2010. We have continued the process and included new studies published up to December 31, 2016. If new interventions not reviewed in prior EBM publications were identified, further searches were made retrospectively to include all appropriate studies.^3^

The methodology used was the same as in prior reports.^2^, ^4^, ^5^ We performed literature searches using electronic databases (Medline, Cochrane Library) and systematic checking of references from review articles and other reports. Inclusion criteria for studies were pharmacological, surgical, and nonpharmacological interventions to treat NMS in PD, commercially available in at least 1 country, assessed using level I, randomized controlled trials (RCTs), where NMS were the primary endpoint measured with an established rating scale or well‐described outcome. The included studies had to have a minimum of 20 patients who were treated for a minimum of 4 weeks. Each study was rated by at least 2 study group members using the Rating Scale for Quality of Evidence^6^ that assigns a percentage rating to the study based on the number of applicable quality criteria fulfilled. Thus, for a study to be designated high quality, it must achieve a quality score of 75% or greater. Each intervention was then assigned an efficacy conclusion—efficacious, likely efficacious, unlikely efficacious, nonefficacious, or insufficient evidence—according to the level of evidence (Supplementary Table e1).^1^ Safety was assessed and assigned as one of the following: acceptable risk with no specialized monitoring, acceptable risk with specialized monitoring, unacceptable risk, or insufficient evidence. The overall implications for clinical practice were then assessed and classed as clinically useful, possibly useful, unlikely useful, not useful, or investigational. In several instances, NMS treatment efficacy conclusions based on RCTs in PD remain inconclusive for agents with proven efficacy in the same condition outside of PD. We decided, therefore, since the last EBM review in 2011, to categorize those interventions where a signal of efficacy in PD is extrapolated by proven efficacy and license outside of PD as also being possibly useful for PD patients. Indeed, the definition of the implications for clinical practice allows such a procedure.

In this article, we use the terms *negative* and *positive* when referring to adequately powered trials designed to test a well‐specified statistical hypothesis; we understand positive to signify a trial where the primary endpoint was met at the defined level of significance and negative to signify a trial that failed to meet the predefined primary endpoint. Each intervention was considered for the indications as outlined in Table 1.

**Table 1:** Indications of nonmotor symptoms covered by this review

Neuropsychiatric symptoms Depression and depressive symptoms Anxiety and anxiety symptoms Apathy Psychosis Impulse control and related disorders Dementia Cognitive impairment (other than dementia; mainly mild cognitive impairment)
Autonomic dysfunction Drooling Orthostatic hypotension Urinary dysfunction Erectile dysfunction Gastrointestinal dysfunction Excessive sweating
Disorders of sleep and wakefulness Sleep fragmentation and insomnia Rapid eye movement sleep behavior disorder Excessive daytime sleepiness
Others Pain Fatigue Olfactory dysfunction Ophthalmologic dysfunction

## Results and Conclusions

There were no RCTs that met inclusion criteria for the treatment of anxiety disorders, rapid eye movement (REM) sleep behavior disorder (RBD), excessive sweating, or olfactory or ophthalmologic dysfunction. For the treatment of NMS, 37 new studies^3^, ^7^, ^8^, ^9^, ^10^, ^11^, ^12^, ^13^, ^14^, ^15^, ^16^, ^17^, ^18^, ^19^, ^20^, ^21^, ^22^, ^23^, ^24^, ^25^, ^26^, ^27^, ^28^, ^29^, ^30^, ^31^, ^32^, ^33^, ^34^, ^35^, ^36^, ^37^, ^38^, ^39^, ^40^, ^41^, ^42^ qualified for review; the updated conclusions, according to indication, are presented in Tables 2 to 10 (interventions with new studies published since January 2011 or prior to this date in the case of newly identified interventions not previously reviewed are indicated in bold and changes in conclusions are italicized). We excluded trials that did not fulfill the inclusion criteria for review^39^, ^43^, ^44^, ^45^, ^46^, ^47^, ^48^, ^49^, ^50^, ^51^, ^52^, ^53^, ^54^, ^55^, ^56^, ^57^, ^58^ and where NMS were not an inclusion criterion, that is, where NMS did not represent a PD‐specific indication.^59^, ^60^, ^61^, ^62^, ^63^, ^64^, ^65^, ^66^, ^67^, ^68^, ^69^, ^70^, ^71^, ^72^, ^73^, ^74^, ^75^, ^76^, ^77^, ^78^, ^79^, ^80^ Unless otherwise specified, safety conclusions are “acceptable risk without specialized monitoring.”

**Table 2:** Interventions to treat depression, including depressive symptoms in PD

Intervention				
Drug class/ intervention strategy	Drug/intervention	Efficacy	Safety	Practice implications
Dopamine Agonists	Pramipexole	Efficacious	Acceptable risk without specialized monitoring	Clinically useful
	Pergolide	Insufficient evidence	Acceptable risk with specialized monitoring	Not useful
	**Rotigotine**	*Unlikely efficacious*	*Acceptable risk without specialized monitoring*	*Investigational*
Monoamine oxidase B (MAO‐B) inhibitors	**Rasagiline**	*Insufficient evidence*	*Acceptable risk without specialized monitoring*	*Investigational*
	Selegeline	Insufficient evidence	Acceptable risk without specialized monitoring	Investigational
	Moclobemide	Insufficient evidence	Acceptable risk with specialized monitoring^a^	Investigational
Tricyclic antidepressants	Nortriptyline	Likely efficacious	Acceptable risk without specialized monitoring^b^	Possibly useful
	Desipramine	Likely efficacious	Acceptable risk without specialized monitoring^b^	Possibly useful
	Amitriptyline	Insufficient evidence	Acceptable risk without specialized monitoring^b^	*Possibly useful* c
Selective serotonin reuptake inhibitors/selective serotonin norepinephrine reuptake inhibitors	Citalopram	Insufficient evidence	Acceptable risk without specialized monitoring^e^	*Possibly useful* d
	Sertraline	Insufficient evidence	Acceptable risk without specialized monitoring^e^	*Possibly useful* d
	**Paroxetine**	insufficient evidence	Acceptable risk without specialized monitoring^e^	*Possibly useful* d
	Fluoxetine	Insufficient evidence	Acceptable risk without specialized monitoring^e^	*Possibly useful* c
	**Venlafaxine**	*Efficacious*	*Acceptable risk without specialized monitoring* e	*Clinically useful*
Other antidepressants	Atomoxetine	Insufficient evidence	Acceptable risk without specialized monitoring	Investigational
	Nefazodone	Insufficient evidence	Unacceptable risk	Not useful
Alternative therapies	'Ω‐3 fatty acids	Insufficient evidence	Acceptable risk without specialized monitoring	Investigational
Nonpharmacological interventions	**rTMS**	*Insufficient evidence*	*Acceptable risk without specialized monitoring* f	*Possibly useful (short term)*
	**CBT**	*Likely efficacious*	*Insufficient evidence* g	*Possibly useful*

CBT, cognitive‐behavioral therapy; RCTs, randomized controlled trials; SSRI, selective serotonin reuptake inhibitors; TCA, tricyclic antidepressants.

a Combined treatment with either TCAs or SSRIs carries an unacceptable risk.

b Typical antimuscarinic adverse events have to be considered, such as dry mouth, constipation, urinary retention, and hyperhidrosis. Moreover, concomitant treatment of PD patients with TCAs can contribute to psychosis, sedation, and daytime sleepiness as well as to cognitive dysfunction or delirium when used in patients with PD dementia.^2^ The risk of mortality has to be considered if overdosing occurs. TCAs should be used with caution in patients with a history of urinary retention, angle‐closure glaucoma, increased intraocular pressure, cardiovascular disorders, and cognitive dysfunction. Of all the antidepressants, available data indicate that TCAs and citalopram at higher dosages pose the greatest risk for QT interval; Monoamine oxidase B prolongation in older adults.^176^

c Although RCTs did not contain a placebo arm, the practice implication is ““possibly useful” because of proven antidepressant efficacy and license outside of PD.

d Although RCTs for PD depression report conflicting data for efficacy, the practice implication is “possibly useful” because of proven antidepressant efficacy and license outside of PD.

e There are concerns about the induction of the serotonin syndrome when used in conjunction with the MAO‐B inhibitors selegiline and rasagiline.^2^ Hyponatremia may be associated with SSRI use, especially in elderly people with low body weight and concomitant use of diuretics, thought to be secondary to the development of the syndrome of inappropriate antidiuretic hormone.^2^ Of all the SSRIs available, data indicate that citalopram at higher dosages poses the greatest risk for QT prolongation in older adults (aged 60 years and above),^176^ such as regular electrocardiograph monitoring should be performed with citalopram when prescribed at a dose >20 mg/day in elderly patients.

f The FDA notes that labeling should include precautions for the use of rTMS devices in the treatment of patients with depressive or related conditions where safety and efficacy have not been established such as in movement disorders.^177^

g In general, the reporting of adverse events in CBT trials is limited;^85^, ^86^ in most behavioral health clinical trials, there is a lack of monitoring of adverse events, including serious adverse events such as suicide attempts, completed suicides, and psychiatric hospitalizations.^86^ Temporary increases in anxiety during behavioral health clinical trials are often considered a normal part of therapy and are therefore not documented as possible adverse events.^86^

**Table 3:** Interventions to treat apathy in PD

Intervention				
Drug class/intervention strategy	Drug/intervention	Efficacy	Safety	Practice implications
Dopamine agonists	**Piribedil** a	*Likely efficacious*	*Acceptable risk without specialized monitoring*	*Possibly useful*
	**Rotigotine**	*Unlikely efficacious*	*Acceptable risk without specialized monitoring*	*Investigational*
Acetylcholinesterase inhibitors	**Rivastigmine**	*Efficacious*	*Acceptable risk without specialized monitoring* b	*Possibly useful*

a Recommendations apply only for PD patients following STN stimulation.

b Worsening of tremor may occur in some patients treated with cholinesterase inhibitors. Medical monitoring for cholinergic effects could include blood pressure or electrocardiograph monitoring but acetylcholinesterase inhibitors are considered to pose an acceptable risk even without specialized monitoring.^2^

**Table 4:** Interventions to treat impulse control and related disorders in PD

Intervention		Efficacy	Safety	Practice implications
Drug class/intervention strategy	Drug/intervention			
N‐methyl‐D‐aspartate (NMDA) antagonists	Amantadine^a^	Insufficient evidence	Acceptable risk without specialized monitoring	Investigational
Anti‐opioids	**Naltrexone** b	*Insufficient evidence*	*Insufficient evidence*	*Investigational*
Nonpharmacological interventions	**CBT** b	*Likely efficacious*	*Insufficient evidence* c	*Possibly useful*

CBT, cognitive‐behavioral therapy**.**

a Recommendations apply for PD patients with pathological gambling.

b Recommendations apply for PD patients with impulse control disorders.

c See Table 2.

**Table 5:** Interventions to treat dementia and nondementia cognitive impairment in PD

Intervention				Practice implications
Drug class/intervention strategy	Drug/intervention	Efficacy	Safety	
**Dementia**				
Acetylcholinesterase inhibitors	**Donepezil**	Insufficient evidence	Acceptable risk without specialized monitoring^a^	*Possibly useful* b
	**Rivastigmine**	Efficacious	Acceptable risk without specialized monitoring^a^	Clinically useful
	Galantamine	Insufficient evidence	Acceptable risk without specialized monitoring^a^	*Possibly useful* c
N‐methyl‐D‐aspartate (NMDA) antagonists	Memantine	Insufficient evidence	Acceptable risk without specialized monitoring	Investigational
**Nondementia cognitive impairment**				
Acetylcholinesterase inhibitors	**Rivastigmine**	*Insufficient evidence*	*Acceptable risk without specialized monitoring* d	*Investigational*
Monoamine oxidase B (MAO‐B) inhibitors	**Rasagiline**	*Insufficient evidence*	*Acceptable risk without specialized monitoring*	*Investigational*
Nonpharmacological Interventions	**Transcranial direct‐current stimulation (T‐DCS)**	*Insufficient evidence*	*Insufficient evidence*	*Investigational*
	**Cognitive rehabilitation**	*Insufficient evidence*	*Insufficient evidence*	*Investigational*

RCTs, randomized controlled trials.

a See Table 1.

b Refers to donepezil 10 mg; although RCTs to treat dementia in PD with donepezil report conflicting data for efficacy, the practice implication for donepezil is “possibly useful” because of the proven antidementive efficacy and license outside of PD.

c Although there is “insufficient evidence” for galantamine to be rated for the treatment of dementia in PD, the practice implication is “possibly useful” because of the proven antidementive efficacy and license outside of PD. Moreover, there were positive signals in favor for galantamine in the trial performed for PD dementia.

d See Table 3.

**Table 6:** Interventions to treat psychosis in PD

Drug	Efficacy	Safety^a^	Practice implications
Clozapine	Efficacious	Acceptable risk with specialized monitoring	Clinically useful
**Olanzapine**	*Not efficacious*	Unacceptable risk	*Not useful*
Quetiapine	Insufficient evidence	Acceptable risk without specialized monitoring	*Possibly useful* b
**Pimavanserin**	*Efficacious*	*Acceptable risk without specialized monitoring* c	*Clinically useful*

RCTs, randomized controlled trials.

a The FDA mandates that antipsychotic drug manufacturers add black box warnings to labels and prescribing information because of the link found between antipsychotics and an increased mortality risk in elderly dementia patients. Moreover, antipsychotic medication may be associated with QT interval prolongation.^178^

b Although there is insufficient evidence for quetiapine to be rated for the treatment of psychosis in PD, the practice implication is “possibly useful.” There are no high‐quality RCTs available for the treatment of quetiapine for psychosis in PD, and quetiapine was similarly efficacious to clozapine in the clozapine‐controlled trials.

c There is a lack of safety data regarding durability beyond 6 weeks. There were more serious adverse events in the pimavanserin arm (7.9%) when compared with the placebo arm (3.5%), but without a unifying pattern and as such it is difficult to interpret these as drug related.^29^ Nevertheless, the FDA has very recently conducted an evaluation of available information about pimavanserin after the publication of reports of postmarketing adverse events.^90^ Based on the analysis of all available data, the FDA did not identify any new or unexpected safety findings with pimavanserin. After a thorough review, the FDA's conclusion remains unchanged that the drug's benefits outweigh its risks for patients with hallucinations and delusions of PD psychosis.^91^ Although the FDA did not identify any new or unexpected safety risks, there should be awareness of the possible adverse effects of pimavanserin including QT prolongation (especially with the concomitant use of other antipsychotic drugs or drugs that can cause QT prolongation) and a potential to cause a paradoxical worsening of symptoms.^142^

**Table 7:** Drugs to treat disorders of sleep and wakefulness in PD

Intervention				
Drug class/intervention strategy	Drug/intervention	Efficacy	Safety	Practice implications
**Insomnia**				
Levodopa	Controlled‐release formulation of levodopa/carbidopa	Insufficient evidence	Acceptable risk without specialized monitoring	Investigational
Dopamine agonists	Pergolide	Insufficient evidence	Acceptable risk with specialized monitoring	Not useful
	Piribedil	Insufficient evidence	Acceptable risk without specialized monitoring	Investigational
	Rotigotine	Likely efficacious	Acceptable risk without specialized monitoring	Possibly useful
Hypnotics	Eszopiclone	Insufficient evidence	Acceptable risk without specialized monitoring^1^	*Possibly useful* a
Melatonin	3‐5 mg	Insufficient evidence	Acceptable risk without specialized monitoring	*Possibly useful* b
	50 mg	Insufficient evidence	Insufficient evidence	Investigational
Nonpharmacological interventions	**Continuous positive airway pressure** c	*Likely efficacious*	*Acceptable risk without specialized monitoring*	*Possibly useful*
**Excessive daytime somnolence and sudden onset of sleep**				
Psychoactive drugs	Modafinil	Insufficient evidence	Insufficient evidence^d^	*Possibly useful* e
	**Caffeine**	*Insufficient evidence*	*Acceptable risk without specialized monitoring*	*Investigational*
Nonpharmacological interventions	**Continuous positive airway pressure** c	*Likely efficacious*	*Acceptable risk without specialized monitoring*	*Possibly useful*

a Although there is insufficient evidence for eszopiclone to be rated for the treatment of insomnia in PD, it can improve global and sleep outcomes for insomnia disorder, and it can be associated with associated with infrequent but serious harms such as fractures and major injury.^179^ Therefore, the practice implication is suggested to be possibly useful.

b Although there is insufficient evidence for melatonin to be rated for the treatment of insomnia in PD, it provided significant benefits on measures of insomnia compared to placebo in patients with PD and insomnia. Moreover, melatonin has not only been approved in the European Union (EU) for patients aged 55 or older suffering from primary insomnia but also has been available over the counter in the United States since the mid‐1990s. Therefore, the practice implication is “possibly useful.”

c Recommendations apply for PD patients with obstructive sleep apnea.

d Rare cases of serious or life‐threatening rash, including Stevens‐Johnson syndrome, toxic epidermal necrolysis, and drug rash with eosinophilia and systemic symptoms have been reported in adults and children in worldwide postmarketing experience. Estimates of the incidence rate for these serious skin reactions in the general population range between 1 to 2 cases per million‐person years. Psychiatric adverse events have been reported in patients treated with modafinil with many, but not all, patients having had a prior psychiatric history; postmarketing adverse events associated with the use of modafinil have included mania, delusions, hallucinations, suicidal ideation, and aggression, some resulting in hospitalization.^2^

e Modafinil provided significant benefits on measures of excessive daytime somnolence when compared with placebo in patients with PD and excessive daytime somnolence^2^ and a recent meta‐analysis of 3 trials evaluating modafinil, which were also included in the previous review,^2^ showed a significant reduction in sleepiness, as assessed by the Epworth Sleepiness Scale.^94^

**Table 8:** Interventions to treat autonomic dysfunction in PD

Symptom	Drug/intervention	Efficacy	Safety	Practice implications
Orthostatic hypotension	Fludrocortisone	Insufficient evidence	Insufficient evidence	*Possibly useful* a
	Midodrine	Insufficient evidence	Insufficient evidence	*Possibly useful* b
	Domperidone	Insufficient evidence	*Acceptable risk with specialized monitoring* c	Investigational
	Yohimbine	Nonefficacious	Insufficient evidence	Investigational
	**Droxidopa** d	*Efficacious (short term)*	Acceptable risk without specialized monitoring (short term)^e^	*Possibly useful*
Sexual dysfunction	**Sildenafil**	*Efficacious*	Acceptable risk without specialized monitoring	*Clinically useful*
Constipation	Macrogol	Likely efficacious	Acceptable risk without specialized monitoring	Possibly useful
	**Lubiprostone**	*Likely efficacious*	*Acceptable risk without specialized monitoring*	*Possibly useful*
	**Probiotics and prebiotic fiber**	*Efficacious*	*Acceptable risk without specialized monitoring*	*Clinically useful*
	**Abdominal massages**	*Insufficient evidence*	*Insufficient evidence*	*Investigational*
Anorexia, nausea and vomiting associated with levodopa and/or dopamine agonist treatment	Domperiodone	Likely efficacious	*Acceptable risk with specialized monitoring* c	Possibly useful
Drooling	Ipratropium Bromide Spray	Insufficient evidence	Insufficient evidence	Investigational
	Glycopyrrolate	Efficacious	Insufficient evidence	Possibly useful
	**Botulinum Toxin B**	Efficacious	Acceptable risk with specialized monitoring	Clinically useful
	Botulinum Toxin A	Efficacious	Acceptable risk with specialized monitoring	Clinically useful
Urinary frequency, urgency, and/or urge incontinence	**Solifenacin** f	*Insufficient evidence*	*Acceptable risk without specialized monitoring* g	*Possibly useful* h

RCTs, randomized controlled trials.

a Although there is insufficient evidence for fludrocortisone to be rated for the treatment of orthostatic hypotension (OH) in PD, it provided some significant benefits in 1 RCT.^2^ Therefore, the practice implication is “possibly useful.”

b Although there is insufficient evidence for midodrine to be rated for the treatment of OH in PD, it provided some significant benefits on measures of OH in RCTs in a mixed population of patients in which only a subgroup had PD.^1^ Therefore, the practice implication is “possibly useful.”

c As a result of the risk of QT interval prolongation and the association with ventricular tachyarrhythmia/sudden cardiac death in PD patients with preexisting cardiac disease.^96^

d Recommendations are for the very short‐term treatment of OH in PD, while there is insufficient evidence to conclude on the efficacy and safety of droxidopa for the treatment of OH in PD for the long term.

e A recent systematic review evaluated the cardiovascular safety of droxidopa in patients with symptomatic neurogenic OH who participated in RCTs (short‐term RCTs: 1 to 2 weeks, n = 444; intermediate RCTs: 8 to 10 weeks, n = 222) and long‐term open‐label studies (n = 422).^97^ Adjusting for exposure time, cardiovascular adverse events rates were 0.30 events/patient‐year in the short‐ and intermediate‐term studies, and 0.15 events/patient‐year in the long‐term open‐label studies, and most evident in patients with preexisting cardiac disorders. Moreover, the risk for supine hypertension has to be considered. Indeed, in the postmarketing surveillance, 1 case with intracranial hemorrhages has been reported.^98^

f For the treatment of overactive bladder.

g A systematic review including 4,188 participants (3,952 participants in placebo‐controlled trials; 650 of them randomized to solifenacin) aged 65 or older randomized to antimuscarinic medications for 4 to 12 weeks and 3,026 randomized to placebo, revealed that treatment for overactive bladder using antimuscarinics in adults aged 65 or older resulted in significant increased risk of several adverse events when compared with placebo including both anticholinergic (eg, dry mouth, constipation) and nonanticholinergic (eg, dyspepsia, dizziness, headaches) adverse events.^101^ Moreover, incidence of urinary tract infections with solifenacin was significantly higher when compared with placebo.

h There were some significant benefits in the active arm and as such the practice implications for solifenacin for the treatment of overactive bladder is “possibly useful” because of the established efficacy and license of solifenacin in this indication outside PD.

**Table 9:** Interventions to treat fatigue in PD

Intervention				
Drug class/intervention strategy	Drug/intervention	Efficacy	Safety	Practice implications
Monoamine oxidase B (MAO‐B) inhibitors	**Rasagiline**	*Efficacious*	*Acceptable risk without specialized monitoring*	*Possibly useful*
Psychoactive drugs	**Methylphenidate**	Insufficient evidence	Insufficient evidence	Investigational
	**Modafinil**	Insufficient evidence	Insufficient evidence^a^	Investigational
Nonpharmacological interventions	**Acupuncture**	*Insufficient evidence*	*Acceptable risk without specialized monitoring*	*Investigational*

a See Table 7.

**Table 10:** Interventions to treat pain in PD

Drug	Efficacy	Safety	Practice Implications
Rotigotine	*Insufficient evidence*	*Acceptable risk without specialized monitoring*	*Investigational*
Oxycodone‐naloxone prolonged release	*Insufficient evidence*	*Acceptable risk without specialized monitoring*	*Possibly useful* a

a There were some significant benefits in the active arm such as the practice implications for oxycodone/naloxone prolonged release for the treatment of pain is “possibly useful” because of the established efficacy and license of oxycodone/naloxone prolonged release in adults with severe chronic pain outside PD.^112^, ^113^

With the exception of 1 low‐quality safety study, which lasted 76 weeks,^32^ all of the studies included in this review lasted 6 months maximum. Therefore, these recommendations do not refer to the long‐term management of a given NMS in PD. Study descriptions and quality scores appear in Supplementary Table e2.

### Treatment of Depression

#### *New Conclusions*

A total of 6 new studies were evaluated.^9^, ^11^, ^16^, ^17^, ^18^, ^19^ We excluded trials not fulfilling the inclusion criteria for review^43^, ^44^, ^55^ and where depression was not an inclusion criterion.^59^, ^60^, ^62^, ^78^ See Table 2 for recommendations.

##### *Tricyclic Antidepressants (TCAs)*

There is “*insufficient evidence”* to make any conclusion on the efficacy of **amitriptyline** for the treatment of depression in PD.^2^ Similar significant benefits were reported in the amitriptyline and sertraline arms of an open‐label randomized trial, which did not include a placebo arm.^2^ Moreover, a recent review on the use of antidepressants for the treatment of major depressive disorder in adults^81^ concluded, based on data from head‐to‐head studies, that amitriptyline was more effective than other antidepressants. The practice implications have been changed so that treatment of depression with TCAs is now considered “*possibly useful.”* 81

##### *Selective Serotonin Reuptake Inhibitors (SSRIS) and Selective Serotonin‐Norepinephrine Reuptake Inhibitors (SSNRIs)*

**Venlafaxine** and **paroxetine** were compared with placebo for the treatment of depression in PD. Both active groups were effective in 1 high‐quality trial;^9^ the practice implications are that **venlafaxine** is “*clinically useful”* for the treatment of depressive symptoms in PD. As a result of conflicting efficacy data of paroxetine for the treatment of depression in PD,^82^ there is still “*insufficient evidence”* for **paroxetine**, as for all SSRIs reviewed. All practice implications have been changed: although studies on the efficacy of **citalopram**, **paroxetine**, and **sertraline** for the treatment of PD depression report conflicting data for efficacy,^2^ and although there were no placebo arms in the studies on **fluoxetine** for the treatment of PD depression,^2^ the practice implications for these SSRIs is that they are “*possibly useful”* because of the established efficacy and license of SSRIs in depression outside PD.^81^ Moreover, some significant benefits were reported in the active arms in the trials performed for depression in PD.^2^ SSRIs, when studied in psychiatric populations, have been found to exhibit an improved safety profile over TCAs with lower incidences of anticholinergic side effects or cardiac arrhythmias. SSRIs may worsen PD tremor in up to 5% of patients and occasionally worsen parkinsonism.^2^ Moreover, citalopram in patients older than age 60 years when using daily doses of more than 20 mg carries the risk of Corrected QT interval (QTc) prolongation such as in these circumstances “specialized monitoring” with regular electrocardiograph monitoring is recommended.

##### *Dopamine Agonists*

One new study evaluated **rotigotine** 19 with negative outcomes and some effects on the primary efficacy analysis in post hoc analyses; the efficacy conclusion is “*unlikely efficacious”* and the practice implication is “*investigational”* for the treatment of depression in PD.

##### *Monoamine oxidase B (MAO‐B) inhibitors*

One new study evaluated **rasagiline** 16; the efficacy conclusion is “*insufficient evidence,”* and as there were significant benefits over the short term, the practice implication is “*investigational.”*

#### *Nonpharmacological Interventions*

##### *Repetitive transcranial stimulation (rTMS)*

rTMS was evaluated in 2 new high‐quality studies for the treatment of depression in PD, which were discrepant regarding depression outcome.^17^, ^18^ Therefore, there is insufficient evidence for rTMS to be rated for the treatment of depression in PD. There is growing evidence that rTMS is efficacious for the treatment of depression in the general population,^83^, ^84^ and it was approved by the Food and Drug Administration (FDA) in 2008 for the treatment of major depressive disorder. Moreover, some beneficial effects on the different depression outcome measures used in the different trials have been reported in PD patients.^2^, ^18^ Therefore, the practice implication is “*possibly useful,”* although it should be kept in mind that the treatment effect is short term, and treatment would need to be repeated at regular intervals.

##### *Cognitive‐behavioral therapy (CBT)*

**CBT** 11 was evaluated in 1 high‐quality positive study. All studies in this field, however, suffer an unavoidable risk of bias because double‐blinding is not possible and so replication of these efficacy results is required. Therefore, CBT can only be rated “*likely efficacious”* for the treatment of depression in PD, and the practice implication is “*possibly useful.”* In general, reporting of adverse events (AEs) in CBT trials is limited (Table 2).^85^, ^86^ Therefore, there is “*insufficient evidence”* to conclude on the safety of CBT in PD patients with depression.

### Treatment of Apathy

#### *New Conclusions*

A total of 3 studies^21^, ^22^, ^23^ were evaluated. We excluded a trial where apathy was not an inclusion criterion.^62^ See Table 3 for recommendations.

##### *Acetylcholinesterase Inhibitors*

**Rivastigmine** 21 was evaluated in 1 positive, small‐sized, high‐quality study. The efficacy conclusion is “*efficacious”* for the treatment of apathy in PD. Because of the small sample size, the practice implication is “*possibly useful.”*

##### *Dopamine Agonists*

**Piribedil** was evaluated in 1 positive, small‐sized, high‐quality study^22^ in PD patients following subthalamic nucleus (STN) deep brain stimulation (DBS) and initial withdrawal of dopamine agonist treatment. The efficacy conclusions is “*likely efficacious”* for the treatment of apathy in PD following STN stimulation with a practice implication of “*possibly useful.”*

One high‐quality trial on **rotigotine** had negative outcomes with some effects on post hoc analyses,^23^ and thus the efficacy conclusion is “*unlikely efficacious”* and the practice implication “*investigational.”*

### Treatment of Impulse Control and Related Disorders

#### *New Conclusions*

A total of 2 new studies^8^, ^31^ were evaluated. Trials not fulfilling the inclusion criteria for review were excluded.^45^ See Table 4 for recommendations.

##### *Opioid Antagonists*

A new, negative, high‐quality study evaluated **naltrexone**.^8^ As there were significant benefits in the active arm, there is “*insufficient evidence”* to conclude on the efficacy of naltrexone for the treatment of impulse control disorders, the practice implication is “*investigational,”* and “*insufficient evidence”* to make any conclusions on its safety.

##### *CBT*

**CBT** was evaluated in 1 low‐quality, positive study,^31^ the efficacy conclusion is “*likely efficacious,”* and the practice implication is “*possibly useful”* for the treatment of impulse control disorders in PD. There is “*insufficient evidence”* on the safety of CBT in PD patients with impulse control disorders (see Treatment of Depression).

### Treatment of Dementia

#### *New Conclusions*

One new study^14^ fulfilled the inclusion criteria for review. In addition, 1 new, open‐label, randomized study evaluated the long‐term safety of rivastigmine capsules versus patches in PD dementia.^32^ See Table 5 for recommendations.

##### *Acetylcholinesterase Inhibitors*

A high‐quality, randomized, open‐label, long‐term safety study of rivastigmine capsules versus patches in PD dementia reported no new safety concerns. A new high‐quality study on the use of donepezil for the treatment of dementia in PD^14^ was negative on the coprimary endpoints. Therefore, there is still “insufficient evidence” for the acetylcholinesterase inhibitors donepezil and galantamine for the treatment of dementia in PD. Practice implications have been changed since the previous review. Some significant benefits were reported in the active arms in the trials performed for PD dementia,^2^, ^14^ and a recent meta‐analysis including the studies reviewed previously revealed that cholinesterase inhibitors slightly improve global impression and enhance cognitive function.^87^ Moreover, because of the established efficacy and license of donepezil and galantamine outside dementia in PD, the practice implications for **donepezil** and **galantamine** are “*possibly useful.”*

### Treatment of Nondementia Cognitive Impairment

#### *New Conclusions*

A total of 5 studies^7^, ^33^, ^34^, ^35^, ^36^ were evaluated. We did not consider clinical trials where cognitive dysfunction was not an inclusion criterion,^59^, ^64^, ^65^, ^66^, ^67^, ^68^, ^69^, ^70^, ^71^, ^72^ where cognition was not the primary endpoint,^46^, ^48^ or those that were post hoc analyses.^47^ See Table 5 for recommendations.

##### *Acetylcholinesterase Inhibitors*

Based on a high‐quality, negative study^36^ with some trend effects and significant benefits in the rivastigmine arm when compared with placebo, and the lack of other RCTs, there is “*insufficient evidence”* to conclude on the efficacy of **rivastigmine** for the treatment of cognitive impairment in PD; practice implications are “*investigational*.”

##### *MAOB Inhibitors*

**Rasagiline** was evaluated in 1 positive, low‐quality, exploratory study^33^ and 1 negative, high‐quality study^7^; therefore, there is “*insufficient evidence”* to conclude on the efficacy of rasagiline for the treatment of cognitive impairment in PD, and the practice implication is “*investigational*.”

##### *Nonpharmacological Interventions*

Active **transcranial Direct Current Stimulation (t‐DCS)** over the left dorsolateral prefrontal cortex versus sham t‐DCS was evaluated for improving cognitive impairment in PD patients receiving computer‐based cognitive training in 1 low‐quality study^34^; therefore, despite significant effects, the efficacy conclusion is “*insufficient evidence,*” and the practice implication “*investigational*.” No safety data were reported in this study, and reports on the use of t‐DCS in PD are scarce^88^; there is, therefore, “*insufficient evidence*” to conclude on the safety of t‐DCS in PD, even though a recent systematic review^88^ found little evidence to suggest that repeated sessions of active t‐DCS pose increased risk when compared with sham t‐DCS within the limits of parameters currently used.

One low‐quality, exploratory study^35^ evaluated **cognitive rehabilitation** for improving cognitive impairment in PD patients receiving computer‐based cognitive training; some significant effects were reported. Because of the exploratory character of the study and the small sample size, the efficacy conclusion is “*insufficient evidence*.” Because of the limited data available for MCI in PD,^34^ the practice implication is “*investigational*.” Because of the lack of safety data,^34^, ^89^ there is “*insufficient evidence*” to conclude on the safety of cognitive rehabilitation for cognitive impairment in PD.

### Treatment of Psychosis

#### *New Conclusions*

A total of 3 new studies^3^, ^29^, ^37^ were evaluated. Trials not fulfilling the inclusion criteria for review were excluded.^49^ See Table 6 for recommendations. Although there is insufficient evidence for **quetiapine** to be rated for the treatment of psychosis in PD, practice implications have been changed since the previous review.^2^ There are no high‐quality RCTs available for quetiapine for the treatment of psychosis in PD; quetiapine was similarly efficacious to clozapine in a clozapine‐controlled trial that did not include a placebo arm.^2^ Therefore, the practice implication is “*possibly useful*” for the treatment of psychosis in PD.

**Olanzapine** was evaluated in a low‐quality, negative study,^37^ as such the conclusions are “*non‐efficacious”* and “*not useful.”*

**Pimavanserin**, a selective serotonin 5‐HT_2A_ inverse agonist without dopaminergic, adrenergic, histaminergic, or muscarinic affinity, was evaluated in 2 level I studies.^3^, ^29^ Although the larger high‐quality study had a positive outcome for antipsychotic efficacy,^29^ the smaller low‐quality study reported a negative outcome for the primary antipsychotic endpoint,^3^ although there were several significant antipsychotic effects in the active arm. Moreover, it was unclear if the primary endpoint was motor safety or antipsychotic efficacy.^3^ Indeed, the study was powered for motor function and as such may have been underpowered for antipsychotic efficacy. Therefore, pimavanserin is considered “*efficacious”* over the short term of 6 weeks for the treatment of psychosis in PD. Although there were no safety concerns, there is a lack of controlled safety data beyond 6 weeks of treatment. Nevertheless, a very recent FDA analysis found no new or unexpected safety risks associated with pimavanserin^90^, ^91^ (Table 6). Therefore, pimavanserin is considered “*clinically useful”* for the treatment of psychosis in PD.

Generally, all atypical antipsychotics must be used with great caution in demented patients with psychosis because of risk of AEs that include falls, cognitive worsening, pneumonia, cardiovascular effects, stroke, and death.^92^

### Treatment of Disorders of Sleep and Wakefulness

#### *New Conclusions*

A total of 3 new studies^41^, ^42^, ^93^ were evaluated. We excluded trials not fulfilling the inclusion criteria for review^51^, ^54^ and where disorders of sleep and wakefulness were not an inclusion criterion.^77^ See Table 7 for recommendations. Although there is “insufficient evidence” for the efficacy of **eszopiclone** and **melatonin** for the treatment of insomnia in PD,^2^ practice implications have been changed since the previous review: both eszopiclone and melatonin have been reported to significantly improve some of the clinical measures of insomnia when compared with placebo in patients with PD and insomnia.^2^ Therefore, the practice implication is “*possibly useful”* for both drugs.

Although there is “insufficient evidence” to conclude on the efficacy of **modafinil** in the treatment of excessive daytime somnolence and sudden onset of sleep in PD,^2^ the practice implications for modafinil for the treatment of insomnia have been changed since the previous review^94^ with the practice implication “*possibly useful*.” Indeed, a recent meta‐analysis of 3 trials evaluating modafinil, which were also included in the previous review,^2^ showed a significant reduction in sleepiness, as assessed by the Epworth Sleepiness Scale.^94^

Based on a low‐quality, positive study,^42^ continuous positive airway pressure therapy is considered “*likely efficacious*” and “*possibly useful*” in improving sleep and daytime sleepiness in patients with PD and obstructive sleep apnea. No safety concerns were identified in this study, and given its wide availability,^95^ continuous positive airway pressure therapy is considered safe with an “*acceptable risk without specialized monitoring*.”

**Caffeine** has been evaluated for the treatment of daytime sleepiness in PD in a high‐quality, negative study.^15^ There were some significant effects for caffeine when compared with placebo, and as such the efficacy conclusion is “*insufficient evidence*” and the practice implication “*investigational*.” Given its wide availability and over‐the‐counter use in many countries, caffeine can be used with an “*acceptable risk without specialized monitoring*.”

##### *Dopamine Agonists*

Based on a low‐quality, negative study^41^ with some significant benefits in the **piribedil** arm and the lack of further RCTs, there is “*insufficient evidence”* to conclude on the efficacy of piribedil for improving vigilance and cognitive performance in those experiencing excessive daytime sleepiness (EDS) while being treated for PD with the oral dopamine agonists pramipexole or ropinirole and who have been switched overnight from their oral dopamine agonists to an equivalent dose of piribedil. The practice implication is “*investigational*.” Based on a low‐quality, positive study, **rotigotine** is “*likely efficacious*” and “*possibly useful*” in improving sleep as it has shown to have significant effects on sleep quality and maintenance in patients with PD.^20^

### Treatment of Orthostatic Hypotension (OH)

#### *New Conclusions*

A total of 2 publications^27^, ^28^ based on data from 1 trial were evaluated. We excluded trials not fulfilling the inclusion criteria for review.^57^ See Table 8 for recommendations. Although there is “insufficient evidence” for the efficacy of midodrine and fludrocortisone for the treatment of OH in PD,^2^ practice implications for the treatment of OH have changed since the previous review. Midodrine provided significant benefits on measures of OH in RCTs in a mixed population of patients of which only a subgroup had PD,^1^ and there were also some significant benefits for fludrocortisone.^2^ Therefore, the practice implications for both **midodrine** and **fludrocortisone** are “*possibly useful*.” Safety conclusions for domperidone have been changed to “*acceptable risk with specialized monitoring*” because domperidone may cause QT prolongation and is associated with increased risk of ventricular tachyarrhythmia and sudden cardiac death in PD patients with preexisting cardiac disease.^96^

**Droxidopa**, a norepinephrine prodrug, was evaluated in a high‐quality trial that was originally designed to evaluate the clinical efficacy of droxidopa during an 8‐week double‐blind period.^27^, ^28^ Because of a preplanned interim efficacy analysis that did not demonstrate a significant difference across groups in the trial's original primary efficacy measure (ie, change in orthostatic hypotension questionnaire (OHQ) composite score),^27^ the original study was stopped for futility and, subsequently, a corresponding change in the trial's primary efficacy measure was undertaken.^28^ Based on this trial, droxidopa is “*efficacious”* for the short‐term treatment of OH in PD, whereas there is “*insufficient evidence”* to conclude on the efficacy of droxidopa for the treatment of OH in PD beyond 1 week. Practice implications are therefore “*possibly useful*.” There were no safety concerns. The RCTs using droxidopa for neurogenic OH were consistent in showing good tolerability of droxidopa.^97^ As for midodrine and fludrocortisone, the risk of supine hypertension has to be considered for droxidopa.^98^ Therefore, droxidopa is considered to pose an “*acceptable risk without specialized monitoring*” during the short term, whereas there is “*insufficient evidence*” to conclude on the safety of droxidopa for the treatment of OH during the long term.

### Treatment of Urinary Dysfunction

#### *New Conclusions*

A total of 1 study^38^ was evaluated for the treatment of urinary dysfunction in PD, and 1 trial not meeting inclusion criteria was excluded.^50^ See Table 8 for recommendations.

**Solifenacin** for the treatment of overactive bladder was evaluated in a high‐quality, negative study.^38^ Because there were some significant benefits in the active arm, there is “*insufficient evidence”* to make a conclusion on efficacy. The practice implications for solifenacin for the treatment of overactive bladder is “*possibly useful”* as there were some significant benefits in this trial^38^ and because of the established efficacy and license of solifenacin in this indication outside PD.^99^, ^100^ No safety concerns were reported. Systematic reviews reported typical peripheral antimuscarinic AEs in patients treated with solifenacin.^99^, ^100^, ^101^ Because of the data available in the geriatric population, solifenacin is considered to pose an “*acceptable risk without specialized monitoring*.”

### Treatment of Erectile Dysfunction (ED)

#### *New Conclusions*

A total of 1 study^102^ was evaluated for the treatment of ED in PD fulfilling the inclusion criteria for review. See Table 8 for recommendations.

**Sildenafil** was evaluated in 1 high‐quality, positive study^102^ and is considered “*efficacious*” for the treatment of ED in PD, and the practice implication is “*clinically useful*.” There is a lack of safety data for sildenafil in PD patients. Taking into account the data available in the general population,^103^ sildenafil is considered to pose an “*acceptable risk without specialized monitoring*.” OH is common in PD and consequences of sildenafil treatment in this population have not been widely explored. Cautious use is advised therefore in parkinsonian patients with OH.

### Treatment of Drooling

#### *Results*

A total of 1 study^13^ was included for the treatment of drooling in PD. Trials not fulfilling the inclusion criteria for review were excluded.^52^, ^104^ See Table 8 for recommendations.

**Botulinum Toxin B (BoNT‐B)** was evaluated in 1 high‐quality, positive study,^13^ and conclusions remain “efficacious” and “clinically useful.” There were no new safety concerns identified in this study. Generally, botulinum toxin type A (BoNT‐A) and botulinum toxin type B (BoNT‐B) are considered to pose an “acceptable risk with specialized monitoring” of the training of the administration of BoNT‐A and BoNT‐B: they should be administered by well‐trained physicians with access to specialized monitoring techniques.^2^

### Treatment of Gastrointestinal Dysfunction

#### *New Conclusions*

A total of 3 new studies^12^, ^39^, ^40^ were included for the treatment of gastrointestinal dysfunction in PD, and trials not fulfilling the inclusion criteria for review were excluded.^53^, ^56^, ^105^ See Table 8 for recommendations.

Based on a low‐quality, positive trial,^12^ **lubiprostone** is considered “*likely efficacious”* and “*possibly useful”* for the treatment of constipation in PD. There were no safety concerns. There is, however, a lack of safety data of lubiprostone in PD patients, but because of the data available in the general and geriatric population,^106^, ^107^ lubiprostone is considered to pose an “*acceptable risk without specialized monitoring*.” Typical AEs of lubiprostone include nausea, diarrhea, and dyspnea.^106^, ^107^

**Probiotics** and **prebiotic fiber** were evaluated in 1 high‐quality, positive study.^40^ The new conclusions are “*efficacious*” and “*clinically useful*.” There are no safety concerns, and given their wide availability and over‐the‐counter use in many countries, probiotics and prebiotic fiber are considered to pose an “*acceptable risk without specialized monitoring*.”

**Abdominal massages** with lifestyle advice versus lifestyle advice alone were evaluated in 1 low‐quality, negative RCT.^39^ Because there were significant signals in both arms, the conclusions are “*insufficient evidence*” and “*investigational*.” Although abdominal massages should not have AEs,^108^ there have been rare reports of potentially fatal complications with abdominal massages for the treatment of constipation in non‐PD patients such as volvulus, small bowel intramural hematoma, or peripheral embolization.^105^, ^109^, ^110^ Safety was not assessed in this study; therefore, there is “*insufficient evidence”* to conclude on the safety of abdominal massage in PD.

### Treatment of Fatigue

#### *New Conclusions*

A total of 2 studies^24^, ^25^ were evaluated. Trials where fatigue was not an inclusion criterion were excluded.^79^ See Table 9 for recommendations.

##### *MAO‐B Inhibitors*

**Rasagiline** was evaluated in 1 positive, small‐sized, low‐quality study^24^ and is considered “*efficacious*” for the treatment of fatigue in PD. Because of the small sample size, the practice implication is “*possibly useful*.”

##### *Nonpharmacological Interventions*

**Acupuncture** was evaluated in 1 negative, low‐quality study in PD,^25^ thus there is “*insufficient evidence”* to conclude on its efficacy in PD. As there were significant benefits in the active arm, the practice implication for acupuncture is “*investigational*.” There were no safety concerns in this study, and a recent meta‐analysis on the effectiveness and safety of acupuncture combined with levodopa and benserazide for the treatment of PD revealed no safety concerns for the use of acupuncture in patients with PD.^111^ As such acupuncture is considered to pose an “*acceptable risk without specialized monitoring*.”

### Treatment of Pain

#### *New Conclusions*

A total of 2 studies^26^, ^30^ were evaluated. See Table 10 for recommendations.

##### *Oxycodone‐Naloxone Prolonged Release*

Based on a high‐quality, negative trial with some signals in the active arm, there is “*insufficient evidence*” to conclude on the efficacy of **oxycodone**‐**naloxone prolonged release**.^26^ Because oxycodone/naloxone prolonged release is an approved treatment option to consider in adults with severe chronic pain, it is “*possibly useful”* for PD patients with chronic pain.^112^, ^113^ There were no safety concerns in the above study. There is a lack of safety data of oxycodone‐naloxone in PD patients, but because of the data available in the general and elderly populations,^114^ oxycodone‐naloxone is considered to pose an “*acceptable risk without specialized monitoring*.” Typical AEs of oxycodone‐naloxone include dizziness, headache, fatigue, worsening cognitive dysfunction, and gastrointestinal tract symptoms such as nausea, vomiting, and constipation.^115^ Moreover, it has to be considered that the increased incidence and severity of constipation in PD might increase the risk of secondary severe complications such as sigmoid volvulus.

##### *Dopaminergic Agents*

Based on a high‐quality, negative trial with some signals in the active arm,^30^ the conclusions for **rotigotine** are “*insufficient evidence”* and “*investigational”* for the treatment of pain in PD.

## Discussion

The present EBM review summarizes the best available evidence from RCTs published from January 2011 to December 2016. Although we have identified a number of efficacious treatments, for many interventions there is insufficient evidence to make adequate conclusions on their efficacy. Indeed, for several indications further RCTs are required. Safety profiles of most of the interventions reviewed in this update are largely based on studies performed in non‐PD populations without firm evidence of efficacy from RCTs in PD. In the absence of such data, there was insufficient evidence to conclude on the safety for many of the interventions reviewed, except when sufficient safety data were available from geriatric populations, in which cases this was clearly stated. Moreover, we have not listed all potential safety issues of the interventions studied. This is beyond the scope of this EBM review and we therefore refer to the respective information leaflets.

Although common, NMS of PD are frequently missed or undeclared during routine consultations^116^ and well‐performed, large‐scale RCTs for the treatment of the different NMS in PD are lacking: only 66% of the trials included in this EBM review fulfilled criteria to be rated as a high‐quality RCT (see Supplementary Table e2). Moreover, no RCTs met inclusion criteria for the treatment of anxiety disorders, excessive sweating, RBD, and sensory symptoms such as olfactory and ophthalmologic dysfunction.^117^, ^118^, ^119^ Therefore, there is insufficient evidence for the treatment of these indications. EBM conclusions are only 1 component of the final dataset that clinicians must use in making treatment decisions. This is particularly important for the pragmatic treatment of NMS in PD, which become increasingly prevalent and obvious during the course of the illness and are a major determinant of quality of life, progression of overall disability, and nursing home placement.^120^ The usefulness of all EBM reviews in day‐to‐day clinical practice requires integration of level I evidence from well‐conducted RCTs with a number of other factors taken into account before deciding on the best therapy required for an individual patient. These factors include economic influences, local availability of the drug/intervention, local drug approval, physicians’ individual clinical experience and judgment, and other patient‐/medical‐related factors such as side effects and tolerability, comorbidities, and comedications as well as patient preferences that all contribute to the final preferred treatment choice. Therefore, off‐label use of an intervention is also sometimes required in the absence of firm level I evidence for a specific indication when this would benefit the individual patient, but such off‐label use is not without its dangers.

NMS add to the overall burden of parkinsonian morbidity, especially in advanced PD stages. In practice, their management is based on careful assessment of triggering or contributing factors, including a rigorous review of the current antiparkinsonian treatment schedule or polypharmacy with other (eg, centrally active) drugs. This is especially important for the treatment of cognitive dysfunction and psychosis, disorders of sleep–wake cycle regulation, and autonomic dysfunction.

Dopaminergic replacement therapies may have contrasting effects on NMS: some, including dopamine agonists and/or rasagiline, are “possibly useful” or “useful” for the treatment of depression, apathy following STN DBS, insomnia, and fatigue. Indeed, several RCTs included in this review have studied the efficacy of dopaminergic replacement therapies for NMS.^7^, ^16^, ^19^, ^20^, ^22^, ^23^, ^24^, ^30^, ^33^, ^41^, ^80^ In contrast, some NMS such as psychosis, impulse control and related disorders (ICRDs), EDS, or constipation can also be worsened or even induced by dopaminergic agents.^121^ Therefore, adapting the antiparkinsonian drug regime is empirically the first step, if feasible.

The pathophysiology of depression in PD is complex and likely to differ considerably from non‐PD patients, reflecting the widespread brainstem and cortical pathology in PD, with the involvement of several neurotransmitters, including dopaminergic, serotonergic, and noradrenergic systems.^122^ Therefore, treatments used in general psychiatry may not be as effective in PD.^122^ Nevertheless, up to 25% of PD patients are on an antidepressant at any given time, most commonly an SSRI.^123^, ^124^ There is now some evidence for the efficacy of SSRIs for PD depression^9^ as well as for SSNRIs,^9^ tricylic antidepressants,^82^ and the dopamine agonist pramipexole,^125^ which are “useful” or “possibly useful” for this indication. For nonpharmacological treatments, there is evidence for the efficacy of CBT,^11^ and many PD patients with depression may prefer psychotherapy.^126^ Some patients with PD depression may respond to rTMS.^127^ Although not specifically a treatment for PD depression, mood generally improves after patients have DBS surgery, perhaps more so for GPi versus STN lead placement.^128^

Dopaminergic and cholinergic denervation are thought to play an important role in PD‐related apathy.^129^, ^130^ Indeed, rivastigmine has been shown to improve apathy in PD and is “possibly useful,“^21^ whereas the evidence is weaker for dopaminergic therapies.^23^, ^131^ On the other hand, for apathy occurring in the context of STN DBS and postoperative withdrawal of PD medications, dopamine agonists can be considered,^22^, ^132^ and piribedil is "possibly useful" for this indication.

A total of 2 large, randomized controlled cholinesterase inhibitor (ChEI) studies in Parkinson's disease dementia (PDD) have been published, 1 positive study for rivastigmine and the other an equivocal study for donepezil.^2^, ^14^ Although statistically significant, the effects of ChEIs in PD are clinically modest.^133^ Although rivastigmine is “clinically useful” for the treatment of PDD, the other ChEIs are “possibly useful.” ChEI treatment appears to be overall well tolerated in PD, outside of nausea and worsening tremor in some patients. On the other hand, the use of memantine is “investigational.” Regarding management of PD‐MCI or cognitive impairment short of dementia, the evidence is much more limited, for both PD and MCI in the general population with insufficient efficacy evidence for rasagiline and rivastigmine^7^, ^33^, ^36^, ^134^ and an unclear role of DBS surgery.^135^, ^136^ There is preliminary evidence that physical^137^ and cognitive exercise^138^ may be beneficial for cognition in PD, limiting anticholinergic medication use^139^ and treating psychiatric conditions might help with cognition long term, and comorbid vascular diseases (eg, hypertension and diabetes) may prevent or limit vascular disease‐associated cognitive decline.

Dose reductions of antiparkinsonian drugs to a level that will lead to a resolution of psychotic symptoms while maintaining sufficient symptomatic motor control is not always feasible and start of antipsychotic therapy becomes necessary.^140^ Frequently, the treatment of psychosis in PD will include the addition of an antipsychotic agent.^141^ Low‐dosage quetiapine, although not formally established as efficacious in RCTs, can be considered a pragmatic first choice because of its improved safety profile when compared with clozapine. In countries where pimavanserin is available, this may be preferable for the treatment of psychosis in PD as it is considered “efficacious” in this instance. Clozapine is another antipsychotic agent with proven efficacy and should be used in all cases that fail following treatment with quetiapine or pimavanserin, but can also be considered a first‐line option despite onerous weekly blood count monitoring. On the other hand, pimavanserin is a relatively new drug and as such there is a lack of long‐term safety data.^90^, ^142^ A very recent FDA analysis found no new or unexpected safety risks associated with pimavanserin.^91^ All antipsychotics must be used with great caution in demented patients with psychosis because of risk of AEs that include falls, cognitive worsening, pneumonia, cardiovascular effects, stroke, and death.^92^ Recently, preliminary research has shown an increased risk of mortality and morbidity with antipsychotic use in PD patients, too, and not specific to dementia.^143^, ^144^ Additional controlled research is needed to determine if antipsychotics increase mortality risk in PD and if pimavanserin is similar to other antipsychotics in this regard.^145^ Moreover, rivastigmine may be another treatment option for psychotic behavior specifically in patients with PD and dementia based on a post hoc analysis of a large, placebo‐controlled study of rivastigmine in PD dementia that showed improvement of hallucinations on rivastigmine.^146^

It is critical for PD patients to be monitored closely for the development of ICRDs as part of routine clinical care, which ideally would include caregiver reports, because ICRDs may have potentially devastating psychological, social, legal, and economic consequences, including divorce, bankruptcy, incarceration, and attempted suicide.^147^, ^148^, ^149^ ICRDs have been most closely related to the use of dopamine agonists,^150^ therefore, the first step in management is usually to try and reduce the dosage of dopamine agonist therapy; in some cases, total cessation is needed.^147^ This unfortunately is frequently complicated by the development of a dopamine agonist withdrawal syndrome, despite compensatory increases in levodopa dosage.^151^, ^152^ CBT was shown to be effective in 1 small study involving cases of moderate severity and is considered "possibly useful,"^31^ whereas other interventions included in this EBM are investigational.^2^, ^8^ STN DBS coupled with postoperative reduction of dopaminergic medications may be effective in reducing ICRDs in a substantial proportion of patients,^153^, ^154^ but RCTs are not available.

Chewing gum/or sucking on hard candy might provide some relief in PD patients with drooling, as these may stimulate voluntary swallowing.^155^ BoNT‐A and BoNT‐B, which block the release of acetylcholine from nerve endings, have been rated as “clinically useful” on the basis of well‐designed RCTs. Special training is needed for performing the injections and ultrasound guidance may reduce the risk of toxin spread to nearby anatomical structures. Both the parotid and submandibular glands should be injected to achieve the best effects.^156^ Glycopyrrolate, a muscarinic antagonist, has been considered “possibly useful” for the short‐term treatment of drooling.

For the treatment of symptomatic OH, the current drug regimen should be reviewed for possible drug‐induced OH. Even when nonpharmacological methods^57^, ^157^, ^158^ are performed properly, many patients still require pharmacological treatment to improve symptomatic OH.^157^ Droxidopa is “clinically useful” for the short‐term treatment of OH, whereas no data from RCTs in PD are available for longer treatment times. Although there is insufficient evidence for the efficacy of fludrocortisone and midodrine for the treatment of OH in PD, it is considered to be “possibly useful” because of its proven efficacy outside of PD with some signals of efficacy detected in the PD trials.^2^ Recently, the norepinephrine transporter blocker atomoxetine has been shown to increase standing blood pressure and reduce the burden of OH symptoms when compared with placebo in mixed cohorts of patients with neurogenic OH.^157^

Before attempting any treatment for lower urinary tract symptoms, urinary tract infections, prostate disease in men, and pelvic floor disease in women should be ruled out. Solifenacin, a type 3 muscarinic receptor antagonist, is considered “possibly useful” in PD, whereas there are no level I data available in PD patients for other muscarinic receptor antagonists or the selective β3‐adrenoceptor agonist mirabegron.

Management of ED in men with PD should first exclude alternative underlying causes such as drug side effects, depression, prostate disorders, or diabetes. When drug treatment is indicated for ED in men with PD, the oral phosphodiesterase‐5 inhibitor sildenafil is “clinically useful.” Other phosphodiesterase‐5 inhibitors have not been tested in PD with RCTs. Open‐label reports have claimed efficacy of the dopamine agonist subcutaneous (s.c.) apomorphine for ED in patients with PD,^159^ and although RCTs with sublingual apomorphine have shown efficacy in non‐parkinsonian patients with ED, RCTs for apomorphine for the treatment of ED in PD are lacking.

Regarding gastrointestinal dysfunction, the EBM review covers anorexia, nausea, and vomiting associated with dopaminergic therapy such as levodopa and/or dopamine agonist treatment as well as constipation.^1^, ^2^ The dopamine D2 receptor blocking agent domperidone remains “possibly useful” in this indication, although safety conclusions have been changed to “acceptable risk with specialized monitoring” because domperidone may cause potentially life‐threatening electrocardiograph changes.^96^ An alternative could be the use of trimethobenzamide, another dopamine D2 receptor blocker, which seems to reduce nausea/vomiting during the first 8 weeks of apomorphine therapy.^56^ There is a lack of level I evidence to conclude on the efficacy of serotonin 5‐HT_3_ antagonistic antiemetic drugs in reducing nausea/vomiting associated with dopaminergic therapy.^160^ Chronic constipation is usually difficult to treat^2^, ^161^ and lifestyle measures, such as increasing fiber and fluid intake, should always be recommended. The use of probiotics and prebiotic fibers is “clinically useful” for the treatment of constipation in PD. Laxatives are another cornerstone of pharmacological treatment. Polyethylene glycol, also known as macrogol, an osmotic agent that causes water to be retained with the stools, has been rated as “possibly useful” in PD. Lubiprostone, an intestinal chloride secretagogue, which has also been rated “possibly useful” in PD, should be reserved for unresponsive patients.

Because of the multiple causative factors involved, the treatment of PD‐related sleep problems and daytime somnolence is usually complex. Careful history taking—often including information from a spouse or caregiver—is essential in identifying the most likely and relevant underlying causes. Treatment options include optimizing PD therapies to improve nocturnal symptom control or to reduce daytime somnolence, treatment of NMS‐like nocturia, depression or mental dysfunction, and counseling about sleep hygiene as well as the addition of sleep or wakefulness promoting drugs. Rotigotine is “possibly useful” for the treatment of insomnia in PD as are eszopiclone and melatonin. Suspicion of comorbid sleep‐disordered breathing requires polysomnographic verification to decide on the need for continuous positive airway pressure therapy, which is “possibly useful” in improving sleep and daytime sleepiness in patients with PD and obstructive sleep apnea.

Before initiating pharmacotherapy for RBD, potential aggravators should be identified and, if possible, removed, for example, the use of SSRIs, SSNRIs or TCAs, which have been associated with causing or worsening RBD in case reports.^162^, ^163^ Treatment options for RBD include clonazepam or melatonin or a combination of these although there are no RCTs available for the treatment of RBD in PD.^163^ A small, controlled, crossover trial reported a decreased number of RBD episodes as monitored by diaries of bed partners with rivastigmine patch when compared with placebo,^51^ but this study was not included in this review as it did not fulfill the inclusion criteria.

New‐onset EDS or sudden onset of sleep following changes in dopaminergic drug type and dose should raise a suspicion of drug‐induced EDS leading to trials of dose reduction or other medication changes. If EDS appears to be caused by insomnia as a result of PD or comorbid conditions such as sleep apnea or depression, these should be treated accordingly. If this is not feasible, the addition of a wake‐promoting drug such as modafinil may be considered, which is “possibly useful.” Often, treating EDS in PD will involve combinations of these measures.

Based on 2 new studies,^26^, ^30^ for the first time, level I evidence is available for the management of pain in PD, a key unmet need. Although both studies failed to show efficacy for the primary endpoint, there were signals in secondary and post hoc analyses (see Supplementary Table e2). Central, musculoskeletal, and nocturnal pain may respond to oxycodone/naloxone–PR although the risks of using an opiate in PD should be considered and patients monitored closely; therefore, the use of oxycodone/naloxone PR is “possibly useful” for the treatment of pain in PD. In patients with nonmotor fluctuations dominated by pain, rotigotine transdermal patch could be considered, although practice implications are “investigational.” There is a need for level I evidence to determine if strategies of more continuous dopaminergic stimulation, including intrajejunal levodopa infusion and subcutaneous apomorphine infusion^164^ or DBS,^165^ can diminish pain in PD, especially if associated with motor or nonmotor fluctuations. There is also a lack of level I evidence on whether other pharmacological approaches, such as the use of antidepressants (tricyclic agents and serotonin‐norepinephrine reuptake inhibitors) and anticonvulsants (gabapentin and pregabalin)^166^ can reduce pain in patients with PD.

Fatigue is an important specific NMS of PD and can be distinct from EDS as well as depressive state.^167^ Management of fatigue is complex, and there are only a few studies providing a good quality evidence base. At the current time, therefore, rasagiline^24^ is “possibly useful” for the management of fatigue in PD when other secondary causes of fatigue have been excluded, whereas the roles of acupuncture, methylphenidate, and modafinil are “investigational.”

Although treatment of motor symptoms as an indication is standard in PD trial methodology, targeting the NMS burden as an indication has rarely been performed in PD despite NMS burden being a key driver of quality of life in PD.^168^, ^169^, ^170^ There are validated screening tools to address NMS in PD in the clinic setting. These include the NMS Questionnaire, the NMS Scale, and part 1 (Non‐Motor Aspects of Experiences of Daily Living) of the MDS‐UPDRS.^2^, ^80^, ^164^, ^171^, ^172^ However, as the NMS of PD include a multitude of clinical systems derived from complex multineurotransmitter dysfunction involving not just the dopaminergic pathways but also cholinergic, noradrenergic, and serotonergic pathways in the brain,^169^ the use of NMS as a holistic endpoint might be a challenge. Although not included in this EBM review, future clinical trials in PD should attempt to include NMS as a holistic endpoint in addition to validated motor and cognitive outcome measures to ensure nonmotor benefits are not overlooked.

In summary, although RCTs in PD have increasingly involved NMS since the previous update of the MDS EBM review, many nonmotor areas still lack an adequate evidence base of high‐quality studies. The MDS is committed to an ongoing process of updating EBM reviews and to making them current and useful to clinicians. Systematic reviews have become a cornerstone of evidence‐based healthcare, but approximately half are out of date after 5 years.^173^ In addition, the methodology that has been standard for years has limitations (eg, with respect to the lack of strict definitions for implications for clinical practice). Therefore, the MDS is considering changes in methodology, including new assessment tools for grading the evidence,^174^, ^175^ as well as more frequent updates to provide clinicians and investigators with an up‐to‐date evidence base for their treatment decision‐making.

## Author Roles

(1) Research project: A. Conception, B. Organization, C. Execution; (2) Manuscript: A. Writing of the first draft, B. Review and Critique

K.S.: 1A, 1B, 1C, 2A, 2B, literature search on interventions to treat psychosis, dementia, olfactory dysfunction and ophthalmologic dysfunction in PD

K.R.C.: 1A, 1B, 1C, 2A, 2B, literature search on interventions to treat cognitive dysfunction (other than dementia), apathy and fatigue in PD

M.C.: 1A, 1B, 1C, 2B, literature search on interventions to treat disorders of sleep and wakefulness as well as pain

S.H.F.: 1A, 1B, 1C, 2B

R.K.: 1A, 1B, 1C, 2B

S.P.L.: 1A, 1B, 1C, 2A, 2B, literature search on interventions to treat autonomic dysfunction in PD

D.W.: 1A, 1B, 1C, 2A, 2B, literature search on interventions to treat depression/depressive symptoms, anxiety/anxiety symptoms and impulse control and related disorders in PD

C.S.: 1A, 1B, 1C, 2B

Collaborators:

L.M.C.: 1C, 2B

E.M.H.: 1C, 2B

B.H.: 1B, 1C, 2B

S.‐Y.L.: 1B, 1C, 2B

W.P.: 1A, 1C, 2B

A.D.‐T.: 1B, 1C, 2B

**FINANCIAL DISCLOSURES of all collaborators:**

L.M.C. reports no conflict of interest related to this work. L.M.C. receives research support from the Michael J Fox Foundation, has received travel payment from MJFF to MJFF conferences, is a paid consultant to MJFF, receives research support for a clinical trial sponsored by Voyager Therapeutics, received travel payments from Voyager Therapeutics to Investigator meeting, and receives royalties from Wolters Kluwer (for book authorship). E.M.H. and B.H. have nothing to declare. S.‐Y.L. has given talks for and received honoraria from the International Parkinson & Movement Disorder Society, Boehringer Ingelheim, Lundbeck, Medtronic, Novartis, and UCB; and has consulted for Lundbeck. W.P. received personal fees from Abbvie, Allergan, Astra Zeneca, BIAL, Boehringer‐Ingelheim, Boston Scientific, GlaxoSmithKline, Ipsen, Lundbeck, Medtronic, MSD, Merck‐Serono, Merz, Novartis, Orion Pharma, Teva, UCB, and Zambon and received royalties from Wiley Blackwell, Oxford University Press, and Cambridge University Press. A.D.‐T. reports personal fees from Grünenthal and Abbvie.

## Full financial disclosures for the past 12 months

K.S. reports personal fees from Teva, UCB, Lundbeck, AOP Orphan Pharmaceuticals AG, Roche, Grünenthal, and Abbvie; honoraria from the International Parkinson and Movement Disorders Society; research grants from the FWF Austrian Science Fund, Michael J. Fox Foundation, and International Parkinson and Movement Disorder Society outside the submitted work. K.R.C. reports the following: advisory board, AbbVie, UCB, Sunovion, Pfizer, Jazz Pharma, GKC, Bial, Cynapsus, Novartis, Lobsor, Stada, Medtronic, Zambon, Profile, Sunovion; honoraria for lectures, AbbVie,Britannia, UCB, Mundipharma, Zambon, Novartis, Boeringer Ingelheim Neuroderm, Sunovion; grants (Investigator Initiated), Britania Pharmaceuticals, AbbVie, UCB, GKC, Bial; academic grants, EU, IMI EU, Horizon 2020, Parkinson's UK, the National Institute for Health Research (NIHR), PDNMG, EU (Horizon 2020), Kirby Laing Foundation, NPF, and Medical Research Council/United Kingdom (MRC). M.C. has given talks for and has received honoraria from the International Parkinson & Movement Disorder Society and has received honoraria from Zambon. S.F.H. has received consultancy and research funding from Acorda, Avanir, Biotie, Britannia, C2N, Cynapsus, Kyowa, Orion, Paladin, Sunovion, Zambon; honoraria from the International Parkinson and Movement Disorders Society, CHDI, and the American Academy of Neurology; research funding from the Michael J. Fox Foundation for Parkinson's disease research, National Institutes of Health, Parkinson Canada, and Toronto Western Hospital Foundation; and salary from the UHN Department of Medicine Practice Plan. R.K. has received research support from Acorda Therapeutics, Adamas, Bial, Biotie Therapies Inc., Britannia Pharmaceuticals, and Stada and fees for speaking or consulting from AbbVie, AOP Pharma, Bial, Britannia Pharmaceuticals, Grünenthal, UCB, and Zambon. S.P.L. has received honoraria from the International Parkinson and Movement Disorders Society. C.S. has received honoraria from Nestle, vTv Therapeutics, Neurotrope Stealth, and the International Parkinson and Movement Disorders Society. D.W. has received research funding or support from the Michael J. Fox Foundation for Parkinson's Research, National Institutes of Health (NINDS), Novartis Pharmaceuticals, Department of Veterans Affairs, Avid Radiopharmaceuticals, Alzheimer's Disease Cooperative Study, and the International Parkinson and Movement Disorder Society; honoraria from AbbVie, Acadia, Biogen, Biotie (Acorda), Bracket, Clintrex LLC, Eisai Inc., Eli Lilly, Janssen, MedEdicus, Merck, Novartis, Pfizer, Takeda, Teva Pharmaceuticals, UCB, and the CHDI Foundation; license fee payments from the University of Pennsylvania for the Questionnaire for Impulsive‐Compulsive Disorders in Parkinson's Disease (QUIP) and QUIP‐RS; royalties from Wolters Kluwer and; and fees for legal consultation for lawsuits related to medication prescribing in patients with Parkinson's disease.

## Supporting information

Supplementary table e1 Definitions for specific recommendations Goetz C. Movement disorders: official journal of the Movement Disorder Society (2002)1:
Supplementary material: Table e2 Review of studies for non‐motor symptoms in Parkinson's disease – Study descriptions and quality scores

## APPENDIX 1

### Movement Disorders Society Evidence‐Based Medicine in Movement Disorders Committee: Collaborators of the Parkinson's Disease Update on Non‐Motor Symptoms Study Group

Lana Chahine, MD, Department of Neurology, University of Pittsburgh, Pittsburgh, PA, USA

Eva‐Maria Hametner, MD, Department of Neurology, Medical University Innsbruck, Innsbruck, Austria

Beatrice Heim, MD, Department of Neurology, Medical University Innsbruck, Innsbruck, Austria

Shen‐Yang Lim, MD, FRACP, Division of Neurology and the Mah Pooi Soo & Tan Chin Nam Centre for Parkinson's & Related Disorders, University of Malaya, Kuala Lumpur, Malaysia

Werner Poewe, MD, Department of Neurology, Medical University Innsbruck, Innsbruck, Austria

Atbin Djamshidian‐Tehrani, MD, Department of Neurology, Medical University Innsbruck, Innsbruck, Austria
