# Supplementary material for: Update on treatments for nonmotor symptoms of Parkinson's disease—an evidence‐based medicine review
Source: Mov Disord. 2019 Jan 17;34(2):180–98. doi: 10.1002/mds.27602 (PMC6916382; doi:10.1002/mds.27602)
Supplement: Supplementary file 1 — Supplementary table e1 Definitions for specific recommendations Goetz C. Movement disorders: official journal of the Movement Disorder Society (2002)1: Supplementary material: Table e2 Review of studies for non‐motor symptoms in Parkinson's disease – Study descriptions and quality scores [file MDS-34-180-s001.docx]

**Update on Treatments for the Non-Motor Symptoms of Parkinson’s Disease – an Evidence-Based Medicine Review**

**SUPPLEMENTARY TABLES**

**Supplementary table e1.**

**Definitions for specific recommendations Goetz C. Movement disorders : official journal of the Movement Disorder Society (2002)^1^:**

| **Efficacy Conclusions** | **Definition** | **Required Evidence** |
| --- | --- | --- |
| **Efficacious** | Evidence shows that the intervention has a positive effect on studied outcomes | Supported by data from at least one high-quality (score ≥75%) randomized controlled trial without conflicting level I data |
| **Likely efficacious** | Evidence suggests, but is not sufficient to show, that the intervention has a positive effect on studied outcomes | Supported by data from any level I trial without conflicting level I data |
| **Unlikely efficacious** | Evidence suggests that the intervention does not have a positive effect on studied outcomes | Supported by data from any Level-1 trial without conflicting Level-1 data |
| **Non-efficacious** | Evidence shows that the intervention does not have a positive effect on studied outcomes | Supported by data from at least one high-quality (score ≥75%) RCT without conflicting level I data |
| **Insufficient evidence** | There is not enough evidence either for or against efficacy of the intervention in treatment of Parkinson’s disease | All the circumstances not covered by the previous statements |
| **Safety** | | |
| **Acceptable risk without specialized monitoring** | | |
| **Acceptable risk with specialized monitoring** | | |
| **Unacceptable risk** | | |
| **Insufficient evidence to make conclusions on the safety of the intervention** | | |
| **Implications for Clinical Practice** | | |
| **Clinically useful** | For a given situation, evidence available is sufficient to conclude that the intervention provides clinical benefit | |
| **Possibly useful** | For a given situation, evidence available suggests, but is insufficient to conclude that the intervention provides clinical benefit | |
| **Investigational** | Available evidence is insufficient to support the use of the intervention in clinical practice, further study may be warranted | |
| **Unlikely useful** | Available evidence suggests that the intervention does not provide clinical benefit | |
| **Not useful** | For a given situation, available evidence is sufficient to say that the intervention provides no clinical benefit | |

**Supplementary material: Table e2**

**Review of studies for non-motor symptoms in Parkinson’s disease – Study descriptions and quality scores**

**Abbreviations:**

**ACE-R: Addenbrooke's Cognitive Examination-Revised**

**ADAS-cog: Alzheimer's Disease Assessment Scale–Cognitive subscale**

**ADCS-CGIC: Alzheimer’s Disease Cooperative Study—Clinical Global Impression of Change**

**AHI: Apnea-hypopnea index**

**AS: Apathy Scale**

**BDI: Beck Depression Inventory**

**BDI-A: Beck Depression Inventory Amended**

**BDI-II: Beck Depression Inventory II**

**BoNT-B: Botulinum toxin type B**

**BPRS: Brief Psychiatric Rating Scale**

**CBT: Cognitive-behavioral therapy**

**CGI-C: Clinical Global Impression–Change ()**

**CIBIC+: Clinician's Interview-Based Impression of Change Plus Caregiver**

**CPAP: Continuous positive airway pressure**

**CR: Cognitive rehabilitation**

**CT: Cognitive training**

**DIP: Drug-induced psychosis**

**DLPFC: Dorsolateral prefrontal cortex**

**DSM-IV-TR: Diagnostic and Statistical Manual of Mental Disorders, 4th text revision**

**ED: Erectile dysfunction**

**EDS: Excessive daytime sleepiness**

**ESS: Epworth sleepiness scale**

**AE: Adverse event**

**HAM-D: Hamilton Depression Rating Scale**

**HAM-D-17: Hamilton Depression Rating Scale 17-item version**

**H&Y: Hoehn and Yahr**

**IIEF: Erectile Function domain of the International Index of Erectile Function**

**ICBs: Impulse control behaviors**

**ICD: Impulse control disorder**

**ITT: Intention to treat**

**LARS: Lille Apathy Rating Scale**

**MADRS: Montgomery-Asberg Depression Rating Scale**

**MFIS: Modified Fatigue Impact Scale**

**MMSE: Mini-mental state exam**

**nOH: Neurogenic orthostatic hypotension**

**NPI: Neuropsychiatric disturbances**

**OAB: Overactive bladder**

**OHSA: Orthostatic Hypotension Symptom Assessment**

**OHQ: Orthostatic Hypotension Questionnaire**

**OSA: Obstructive sleep apnea**

**OXN-PR: Oxycodone-naloxone prolonged release**

**Penn State Worry Questionnaire (PSWQ)**

**PD: Parkinson’s disease**

**PDD: PD dementia**

**PSG: polysomnography**

# PSQI: Pittsburgh Sleep Quality Index

# rTMS: Repetitive transcranial magnetic stimulation

**SAPS-PD: Schedule for Assessment of Positive Symptoms in PD psychosis**

**SCOPA-COG: Scales for Outcomes of Parkinson’s Disease-Cognition**

**SDMT: Symbol digit Modalities Test**

**STN: Subthalamic nucleus**

**TAP: Test battery for attention performances**

**UPDRS: Unified Parkinson's disease rating scale**

| Intervention | Reference | Investigated population | Sample size | Intervention/comparator | Primary outcome | Main result | Quality score (%) | Safety | Comments |
| --- | --- | --- | --- | --- | --- | --- | --- | --- | --- |
| **INTERVENTIONS TO TREAT NEUROPSYCHIATRIC SYMPTOMS** | | | | | | | | | |
| **INTERVENTIONS TO TREAT DEPRESSION AND DEPRESSIVE SYMPTOMS IN PD** | | | | | | | | | |
| Paroxetine | Richard I. H. Neurology (2012)^9^: | Patients with PD and depressive disorder (Diagnostic and Statistical Manual of Mental Disorders, 4th text revision [DSM IV] or operationally defined subsyndromal depression (presence of ≥ 2 depressive symptoms at threshold or subthreshold levels on the structured clinical interview for DSM-IV, at least one of which had to include depressed mood or anhedonia) and a score of > 12 on the Hamilton Depression Rating Scale (HAM-D -17). | 115 | Randomized to receive paroxetine (max daily dosage 40mg; n=42), venlafaxine XR (max daily dose 225mg; n=34) or placebo (n=39). 56% of those in the placebo arm, 69% of those in the paroxetine arm, and 65% of venlafaxine XR arm had major depression. | Reduction in HAM-D -17 score compared to placebo at 12 weeks. | There was a significant reduction in the HAM-D-17 score relative to placebo for both active treatment arms [paroxetine: 6.2 (97.5%CI 2.2-10.3, p=0.0007); venlafaxine XR: 4.2 (97.5%CI 0.1-8.4, p=0.02)] with no significant difference between active treatment arms (p=0.28). | 86% | There were no safety concerns in this study |  |
| Venlafaxine | Richard I. H. Neurology (2012)^9^: | As above |  |  |  |  |  |  |  |
| Rasagiline | Barone P. European journal of neurology (2015)^16^: | Non-demented (MMSE > 25) PD patients (Hoehn & Yahr [H&Y] 1-3) with depressive symptoms (Beck depression inventory-amended [BDI-IA ≥ 15). | 123 | Patients were randomized 1:1 to receive rasagiline 1 mg daily (n=58) or matching placebo (n=65). | Change from baseline to week 12 in depressive symptoms measured by the BDI-IA total score. | At week 12 there was no significant difference between groups for the reduction in total BDI-IA score (-5.40 ± 0.79 for rasagiline vs. -4.43 ± 0.73; p=0.368), while analysis at week 4 did show a difference in favour of rasagiline (-5.46 ± 0.73 for rasagiline vs. -3.22 ± 0.67 for placebo; p = 0.026). | 87.5% | Four patients in the rasagiline group withdrew due to an adverse event (AE; aggravated dyskinesia, vertigo, left trunk flexion due to PD, nausea) versus none in the placebo group. | Post hoc analysis showed superiority of rasagiline versus placebo in the UPDRS Part I depression item (p = 0.04) |
| Rotigotine | Chung S. J. Expert opinion on pharmacotherapy (2016)^19^: | Patients with early /advanced PD, with depression (BDI-II score ≥ 16), a modified H&Y I–III, without motor fluctuations or dyskinesia, stable motor symptoms for at least 4 weeks prior to screening as judged by the local investigator, and a MMSE ≥ 24. | 380 | Patients were randomized 1:1 to receive rotigotine daily (n=184; mean dose: 7.5±3.37 mg/24 h) or matching placebo (n=196): up to 7 weeks of titration to an optimal dose or maximal dose of rotigotine 8 mg/24 h in early and 16 mg/24 h in advanced PD patients or matching placebo and 8-week maintenance period. | Change from baseline to week 8 of maintenance period in depressive symptoms measured by the HAM-D-17. | No statistically significant difference between the two patient groups (p=0.1286) in the ITT analysis. | 81.8% | Common adverse events with higher incidence with rotigotine: nausea, application/instillation site reactions, vomiting, and pruritus. 41 (10.8%) patients discontinued owing to adverse events (25 rotigotine/16 placebo). | A post hoc analysis of the primary efficacy variable for the completer set showed significant improvement for rotigotine compared to placebo (p= 0.0216). |
| Non-pharmacological interventions | | | | | | | | | |
| Cognitive-behavioral therapy (CBT) | Dobkin R. D. The American journal of psychiatry (2011)^11^: | PD and depression (DSM-IV, 81% with major depression, antidepressant use in 54% of the patients in both groups | 80 | Individually administered CBT vs. clinical monitoring alone. | Reduction in HAM-D-17. | Significant HAM-D reductions in CBT relative to clinical monitoring alone (p<0.0001: mean change from baseline 7.35 from 20.9 for CBT vs. 0.05 from 19.4 for clinical monitoring alone) at week 10 with maintained improvement at week 14. | 88% | Safety was not assessed in this study. | This is the first RCT on CBT for the treatment of depression in PD.Unavoidable risk of bias because double-blinding is not possible. |
| Repetitive Transcranial Magnetic Stimulation (rTMS) | Brys M. Neurology (2016)^17^: | PD and comorbid major depression with >7 on HAM-D. | 61 | Patients were randomized to one of four groups: bilateral M1 stimulation with sham stimulation of the dorsolateral prefrontal cortex (DLPFC), stimulation of the DLPFC and sham M1 stimulation, stimulation of M1 and DLPFC, or double sham. | Difference in the UPDRS III and HAM-D between pretreatment and 4 weeks. | At 4 weeks, there was a significant change in the UPDRS III in the M1 group (-4.9 points) compared to the sham group (-0.3 points; mean difference = -4.6, 95% confidence interval -0.1 to -9.1, t=-2.1, p<0.05). Unexpectedly, the change in the HAM-D was greater in the sham group (-6.1) than in the DLPFC group (-1.4; mean difference=-4.7, 95% CI 0.7-8.7, t=2.4, p<0.05). | 73.5% | There was no significant difference in AEs between the sham and the active groups. One serious AE occurred (ischemic stroke) in a patient receiving active rTMS. | rTMS lasted 2 weeks, while the primary endpoint was evaluated at 4 weeks. |
| rTMS | Makkos A. Neuropsychobiology (2016)^18^: | PD patients with mild-to-moderate depression (DSM-IV-TR), without antidepressant medication over the last two months. | 46 | Patients were randomly assigned to either a real or sham stimulation group. High-frequency rTMS with three hundred impulses on both sides over the primary motor cortex with a frequency of 5 Hz per day was applied for 10 consecutive days. | Differences between baseline and 30 days in BDI and the validated Hungarian version of the Montgomery-Asberg Depression Rating Scale (MADRS) scores. | The MADRS significantly improved at 30 days in the actively stimulated group (17 vs. 7 points, p=0.003), whereas sham stimulation only provided a slight improvement, which was not significantly different to baseline (15 vs. 13 points, p=0.119). BDI total score improved in the actively treated group from a median of 12 points (IQR: 5.18) to 6 points (IQR: 2-10, p<0.001), while it worsened in the sham group (11 vs.12 points). | 77.5% | No rTMS related AEs were observed. | rTMS lasted 10 days, primary endpoint evaluated at 30 days. |
| **INTERVENTIONS TO TREAT APATHY IN PD** | | | | | | | | | |
| Rivastigmine | Devos D. Journal of neurology, neurosurgery, and psychiatry (2014)^21^: | PD with moderate to severe apathy (LARS score ≥ 16 despite optimized dopaminergic therapy). | 30 | Patients randomly assigned 1:1 to receive rivastigmine (transdermal patch of 9.5 mg/day; n=16) or placebo (n=14). Dopaminergic therapy and subthalamic nucleus (STN) stimulation parameters had to be unchanged 3 months before and throughout the study. | Mean change in the Lille Apathy Rating Scale (LARS) score after 6 months. | Compared to placebo, rivastigmine significantly improved the LARS score after 6 months (−11.5 (−15/−7) to −20 (−25/−12) vs. -13.3 (-16/-12) to -13.5 (-15/-12); p=0.034). The adjusted size effect was -0.9. | 95% | No significant differences in tolerability outcomes were observed between groups. | In the 12-month extension phase a significant reduction in symptoms of apathy was observed in patients previously in the placebo group (median LARS score at 18 month: -16(-21/-9); p < 0.05). |
| Piribedil | Thobois S. Brain : a journal of neurology (2013)^22^: | PD presenting with apathy (Starkstein Apathy Scale > 14, or a five point increase with clinically significant apathy) following STN stimulation. | 37 | Patients were randomized 1:1 to received piribedil up to 300 mg per day (n=19; mean dosage 239.2 + 154.8 mg/24 h) or placebo (n=18) for 12 weeks. | Improvement of apathy as assessed by the reduction of the Starkstein Apathy Scale score. | Intention to treat (ITT) analysis demonstrated a reduction in the Starkstein Apathy Scale score by 34.6% for piribedil vs. 3.2% for placebo (p=0.015). | 80% | No significant AEs were observed. The number of premature study dropouts was seven in the placebo and five in the piribedil group (intolerance to hypodopaminergic symptoms (n=4); hallucination (n=1)). |  |
| Rotigotine | Hauser R. A. BMC neurology (2016)^23^: | Patients with PD and PD-associated apathy according to the Unified Parkinson's Disease Rating Scale (UPDRS) I item 4 and patient-rated Apathy Scale (AS). | 122 | Patients were randomized to receive “low-dose” rotigotine (≤6 mg/24 h for early PD [those not receiving levodopa] or ≤8 mg/24 h for advanced PD [those receiving levodopa]), "high-dose" rotigotine (≤8 mg/24 h for early PD or ≤16 mg/24 h for advanced PD) or placebo, and maintained an optimal/maximal dose for 12 weeks (end of maintenance). | The co-primary efficacy variables were the change from baseline to end of maintenance in the (1) AS score as rated by the patient and (2) UPDRS II + III total score. This was an explanatory study, because recruitment was stopped after an interim futility analysis, which was planned after approximately 120 of 450 patients had been randomized. | There were no differences between the three patient groups regarding patient-rated AS. Regarding the UPDRS II + III, rotigotine improved the scores compared to placebo (low-dose, p = 0.005; high-dose, p = 0.015). Secondary efficacy analyses revealed no significant difference of the AS as rated by the caregiver. | 85% | The most frequent AEs in treated patients were application site reactions, somnolence, and nausea. | Post hoc analyses of the 4 apathy items of the NMSS demonstrated improvement in the combined score for both the low- and high-dose rotigotine groups compared with placebo |
| **INTERVENTIONS TO TREAT MEDICATION-RELATED IMPULSE DYSCONTROL AND ABNORMAL REPETITIVE BEHAVIORS IN PD** | | | | | | | | | |
| Naltrexone | Papay K. Neurology (2014)^8^: | PD patients with impulse control disorder (ICD) symptoms (mean age 61.2 (8.5) years; 68% male), taking DAs for >6 months and on stable doses for >1 month prior to study inclusion. | 50 | Patients were randomized 1:1 to receive naltrexone as a flexible dose (50-100 mg/d) to determine the efficacy and tolerability of naltrexone for the treatment of ICDs. | Response based on the Clinical Global Impression–Change (CGI-C) score at 8 weeks. | There was no between-group difference for response status over time using the CGI-C (response rate at week 8: 54.4% (naltrexone) vs. 33.1% (placebo); p = 0.47). | 82.5% | The most common AE was new-onset nausea, which was more common in the naltrexone group (29.2% vs. 0%, p=0.009). This was reported as mild-moderate intensity and did not lead to study discontinuation in any participants. |  |
| CBT | Okai D. Neurology (2013)^31^: | PD patients and associated impulse control behaviors (ICBs) who had failed to remit despite standard measures, including medication changes, being taken. | 45 | Patients were randomly assigned to immediate treatment with a novel cognitive-behavioral therapy (CBT)–based intervention delivered by a nurse therapist (treatment group; n= 28) or a 6-month waiting list (waiting group; n= 17). | The co-primary outcomes were overall symptom severity (CGI-S) and neuropsychiatric disturbances index (NPI) in the patients and carer burden and distress after 6 months (Zarit Burden interview and the total distress score. on the NPI). | Significant improvement in CGI-S in the CBT group vs. controls, from a mean score consistent with moderate to one of mild illness-related symptoms (4.0 (±0.6) to 2.5 (±1.2) vs. 3.7 (±0.61) to 3.5 (±1.2); p=0.004). 75% were improved in the treatment group, vs. 29% on the waitlist. NPI scores improved significantly (26.0 (±18.3) to 16.4 (±14.2) vs. 22.0 (±13.9) to 23.8 (±18.2); p=0.033). Measures of carer burden and distress did not change significantly. | 67.5% | No serious AEs attributable to the intervention were reported in the trial. | There is insufficient evidence on the safety of CBT in PD patients with depression. |
| **INTERVENTIONS TO TREAT DEMENTIA IN PD** | | | | | | | | | |
| Donepezil | Dubois B. Movement disorders : official journal of the Movement Disorder Society (2012)^14^: | PD dementia (PDD). | 550 | Patients were randomized to receive donepezil 5mg, donepezil 10mg, or placebo for 24 weeks. | Co-primary endpoints were the Alzheimer's Disease Assessment Scale–Cognitive subscale (ADAS-cog) mean changes from baseline to week 24 and Clinician's Interview-Based Impression of Change Plus Caregiver Input (CIBIC+) scores at week 24. | The study was negative on the co-primary endpoints. There were non-significant ADAS-cog mean changes from baseline to week 24 (mean difference from placebo: donepezil 5mg: -1.45, 95%CI: -2.90–0.00, p=0.05; donepezil 10mg: -1.45, 95%CI: -3.04–0.15, p=0.076). On the other hand, CIBIC+ scores were significant better versus placebo for donepezil 10mg (p=0.04), but not for donepezil 5mg (p=0.113) (mean change score at week 24: donepezil 5mg 3.7±1.12; donepezil 10mg 3.6±1.29; placebo 3.9±1.27). | 82% | Higher rates of parkinsonian AEs (donepezil 5mg 10.8%; donepezil 10mg 10.4%; placebo 6.9%) as well as tremor (donepezil 5mg 7.2%; donepezil 10mg 7.1%; placebo 2.9%) were noted in donepezil-treated patients, but the difference was not significant, without apparent dose dependency and no impact on the UPDRS motor scale. | There was a significant treatment-by-country interaction as showed by the preplanned primary analysis for ADAS-cog using an ANCOVA model with equal weighting to countries. But there were highly significant treatment benefits for both donepezil arms using post hoc ADAS-cog analysis conducted based on the model without the treatment-by-country interaction term in ANCOVA. |
| Rivastigmine | Emre M. Clinical neuropharmacology (2014)^32^: | 583 PDD patients (mean age 72.3 years; mean MMSE 20.9; mean H&Y 2.7) with a regular contact to a caregiver. | 583 | Patients were randomized 1:1 to rivastigmine capsules (n=295), which were titrated to 6 mg bid during 16 weeks (initiated at 1.5 mg bid and titrated in 3 mg/d increments every 4 weeks to target or highest well-tolerated dose) followed by a 60-week maintenance period. Subjects in the patch arm (n=288) initiated treatment with 4.6 mg/24 h and were titrated to 9.5 mg/24h after 4 weeks followed by a 68-week maintenance period. Dose adjustments and interruptions were permitted. | AEs due to worsening of PD motor symptoms and discontinuation rate due to predefined AEs with capsules. | The incidence of predefined AEs was 36.1% (95% CI, 30.6–41.8) with tremor being the most commonly reported (24.5%; 95% CI, 19.7–29.8) for capsules. Overall, 4.4% (95% CI, 2.4–7.4) of capsule treated patients discontinued due to worsening of PD motor symptoms. | 78.6% | The overall incidence of patients reporting at least one AE was comparable between groups (capsule, 93.2%; patch, 91.3%). A similar proportion of each group discontinued due to AEs (capsule, 27.2%; patch, 24.7%). |  |
| **INTERVENTIONS TO TREAT COGNITIVE DYSFUNCTION IN PD** | | | | | | | | | |
| Rivastigmine | Mamikonyan E. Movement disorders : official journal of the Movement Disorder Society (2015)^36^: | PD with MCI (Windblad criteria for MCI and a Clinical Dementia Rating of 0.5 and an age- and education-corrected DR-2 score < 8). | 28 | Patients were randomized to 10 weeks of treatment with rivastigmine (the initial 4 weeks at 4.6 mg/24 h, the final 6 weeks at 9.5 mg/24 h, with the option to remain at 4.6 mg/24 h if the higher dose was not tolerated) or matching placebo patch. After a 4-week washout period patients returned for the phase 2 baseline visit and received the treatment not administered in phase 1. | Between group differences at the end-of phase Alzheimer’s Disease Cooperative Study—Clinical Global Impression of Change (ADCS-CGIC). | The CGIC response rate demonstrated a trend effect in favor of rivastigmine (regression coefficient for interaction term in linear mixed-effects model = 0.44, F[df]=3.01 [1, 24], p=0.096). For patients with end-of-phase data available, the mean (standard deviation [SD]) end-of-phase study CGIC scores were 3.48 (0.89) for rivastigmine and 3.92 (0.94) for placebo. | 81.6% | No between-group differences were observed in AEs. |  |
| Rasagiline | Hanagasi H. A. Movement disorders : official journal of the Movement Disorder Society (2011)^33^: | Cognitively impaired, non-demented patients with PD (mean age 66.4 yrs, mean H&Y 1.8) receiving stable dopaminergic treatment. | 48 | Patients were randomized to receive rasagiline 1 mg/day (n=23) or placebo (n=25) for 3 months. | This was an exploratory trial and there was no *a priori* defined primary endpoint. |  | 65% | There were no safety concerns in this study |  |
| Rasagiline | Weintraub D. Movement disorders : official journal of the Movement Disorder Society (2016)^7^: | PD patients (H&Y stage I to III), aged 45 to 80 yrs, with MCI and stable dopaminergic therapy (≥ 30 days preceding baseline visit). | 170 | Patients were randomized (1:1) to 24 weeks of treatment with either rasagiline 1 mg/day or placebo, which was added to their current, stable PD therapy. | Mean change from baseline to week 24 on the Scales for Outcomes of Parkinson’s Disease-Cognition (SCOPA-COG) total score. | Change in SCOPA-COG scores were not significantly different in the rasagiline and placebo groups (adjusted mean: 1.6 (standard error (SE) = 0.5) vs. 0.8 (SE = 0.5) points; LS means difference = 0.8; 95% confidence interval: -0.48, 2.05; p=0.22). | 86.8% | The most common AEs in both groups were falls and dizziness. |  |
| Transcranial Direct Current Stimulation (t-DCS) | Biundo R. Brain stimulation (2015)^34^: | PD patients with MCI. | 24 | Patients were randomly allocated to receive cognitive training (CT) plus real t-DCS over the left dorsolateral prefrontal cortex (n=12, 6 men and 1 woman, age 69.1 ± 7.6) or sham t-DCS (n=12, 8 men and 1 woman, age 72.3 ± 4.1). | No pre-defined primary outcome | At the end of week 4 a significant decrement performance for the real t-DCS compared to sham group in attention/executive skills [Written coding test: real vs. sham t-DCS, p< 0.01, Cohen’s d = 1.52] was observed. At week 16 a strong trend for better performance in the real t-DCS compared with sham stimulation arm in the story learning test [real vs. sham t-DCS, p< 0.07, Cohen’s d = 0.9] and immediate memory index [real vs. sham t-DCS, p< 0.07, Cohen’s d = 0.7] was found. | 47.6% | No safety data were reported in this study. |  |
| Cognitive rehabilitation (CR) | Cerasa A. Neurological sciences : official journal of the Italian Neurological Society and of the Italian Society of Clinical Neurophysiology (2014)^35^: | PD patients with predominant deficits in either attention and/or information processing speed, working memory and/or executive functioning. | 20 | Patients were randomized 1:1 to either the CR program or placebo intervention | Pre-defined primary outcome not specified, several cognitive and psychological outcomes assessed. | Considering all cognitive and psychological domains, the CR group showed significant cognitive improvements in the SDMT (Symbol digit Modalities Test) (T-value = 4.1, p-level = 0.04) and the digit span forward (T-value = 9.3, p-level = 0.01). | 54.5% | No safety data were reported in this study. |  |
| **INTERVENTIONS TO TREAT PSYCHOSIS IN PD** | | | | | | | | | |
| Olanzapine | Nichols M. J. F1000Research (2013)^37^: | PD with drug-induced psychosis (DIP). | 23 | Patients were randomized 1:1:1 to placebo or either of the two doses of olanzapine (2.5mg or 5mg), while allowing for clinically realistic dose adjustments of dopaminergic medication. 14 patients completed the study (7 on placebo, 2 on 2.5mg olanzapine, 5 on 5mg olanzapine). | Brief Psychiatric Rating Scale (BPRS) ratings and CGI (Clinical Global Impression) scored from videotaped interviews by an observer blinded to dose assignment and to interview timing. The UPDRS motor subscale was the primary measure of tolerability. | In study completers, ANOVA analysis revealed no significant differences between olanzapine and placebo groups in BPRS psychosis reduction (p=0.536), parkinsonism (p=0.608), or CGI, MMSE. | 73.7% | Side effects were reported by more olanzapine vs. placebo treated subjects (p<0.04), many citing motor worsening (0% of placebo withdrawers vs. 21% of olanzapine withdrawers). |  |
| Pimavanserin | Cummings J. Lancet (2014)^29^: | PD (UK Brain Bank criteria) lasting at least 1 year, age ≥40 yrs and psychotic symptoms that developed after PD diagnosis and were present for at least 1 month, occurred at least weekly, and were severe enough to warrant antipsychotic treatment (Neuropsychiatric inventory items A (delusions) and/or B (hallucinations) combined score >5 or an individual score >4). | 199 | Patients were randomized to receive placebo (n=94) or pimavanserin 40 mg daily (n=105). | Change in total Schedule for Assessment of Positive Symptoms in PD psychosis (SAPS- PD) score from baseline to day 43. | For 90 recipients of placebo and 95 recipients of pimavanserin included in the primary analysis, pimavanserin was associated with a −5.79 (-37%) decrease in SAPS-PD scores compared with −2.73 (-14%) for placebo (difference –3.06, 95% CI –4.91 to –1.20; p=0.001). CGI-S (-1.02 (0.12) vs. -0.44 (0.12); p=0.0007). | 90.5% | Ten patients in the active treatment arm discontinued due to AEs (4 due to psychotic disorder or hallucination within 10 days of start of the study drug) compared with two in the placebo group. No treatment related impairment of motor function (UPDRS) was detected in either group. |  |
| Pimavanserin | Meltzer H. Y. Neuropsychopharmacology : official publication of the American College of Neuropsychopharmacology (2010)^3^: | PD patients with psychosis according to established criteria (Mov Disord 22: 313–318). | 60 | Patients were randomized to receive placebo or pimavanserin. | Unclear if primary endpoint was motor safety or antipsychotic efficacy. | The principal measures of efficacy of antipsychotic response to pimavanserin, the SAPS total domain score, only showed a trend. However, the pimavanserin-treated patients showed significantly greater improvement in some but not all measures of psychosis, including SAPS global measures of hallucinations and delusions, persecutory delusions, and the UPDRS measure of delusions and hallucinations. | 73.7% | Pimavanserin did not differentiate from placebo with regard to motor impairment, sedation, hypotension, or other side effects. | It was unclear if primary endpoint was motor safety or antipsychotic efficacy in this study. Indeed, the study was powered for motor function. Pimavanserin did not differentiate from placebo with regard to motor impairment, sedation, hypotension, or other side effects. |
| **INTERVENTIONS TO TREAT DISORDERS OF SLEEP AND WAKEFULNESS IN PD** | | | | | | | | | |
| Continuous positive airway pressure (CPAP) | Neikrug A. B. Sleep (2014)^42^: | PD and obstructive sleep apnea (OSA) patients (mean age 67.2 ± 9.2 y; 12 females). | 38 | Patients were randomized in a 1:1 ratio into 6 weeks of therapeutic treatment (n=38) or 3 weeks of placebo followed by 3 weeks of therapeutic treatment. | This was an exploratory study including several polysomnography (PSG) outcome measures (sleep efficiency; %sleep stages: N1, N2, N3, R; arousal index, apnea-hypopnea index (AHI); and %time oxygen saturation < 90%: %time SaO2 < 90%) as well as multiple sleep latency test outcome measures (mean sleep-onset latency, MSL and the number of naps on which patients fell asleep in < 10 min.). There was, however, no correction for multiple comparisons of the multiple outcome measures. | Therapeutic CPAP showed significant decrease in AHI (20 (SD 14.3) vs. 5.12(SD 8.1); p=0.01), %time SaO2 < 90% (12.9% (SD 14.9) vs. 1.2% (SD 3.8); p<0.01), %N2 (p=0.048), and significant increase in %N3 (p=0.025) indicating effectiveness of CPAP in the treatment of OSA, improvement in nighttime oxygenation, and in deepening sleep. Therapeutic treatment resulted in significant decreases in arousal index (t=3.4, p=0.002). All improvements after 3 weeks were maintained at 6 weeks. Finally, 3 weeks of therapeutic CPAP also resulted in overall decreases in daytime sleepiness as measured by MSL (p= 0.011) and on the number of naps on which patients fell asleep in < 10 min (p=0.027). | 57.5% | There were no safety concerns identified | This was an exploratory study in PD patients with OSA including several PSG outcome measures (sleep efficiency; %sleep stages: N1, N2, N3, R; arousal index, AHI; and %time oxygen saturation < 90%: %time SaO2 < 90%) as well as MSLT outcome measures (mean sleep-onset latency, MSL and the number of naps on which patients fell asleep in < 10 min.). |
| Caffeine | Postuma R. B. Neurology (2012)15: | PD and excessive daytime sleepiness (EDS) (Epworth sleepiness scale score, ESS ≥10) | 61 | Patients were randomized to receive caffeine 200 mg daily for 3 weeks, followed by 400 mg for another 3 weeks (n=30) or matching placebo (n=31). | The primary endpoint was change in ESS score. | On the primary ITT analysis, caffeine resulted in a not significant reduction in ESS score of -1.71 points (95% CI -3.57, 0.13). | 95% | AEs were comparable in caffeine and placebo groups. |  |
| Piribedil | Eggert K. Clinical neuropharmacology (2014)^41^: | PD patients experiencing excessive daytime sleepiness (ESS ≥10) on pramipexole or ropinirole. | 80 | Patients were randomly assigned either to receive piribedil (n=44; mean daily dose 213.2 mg (61.9)) or to continue their standard therapy (n=36; mean dose of pramipexole 2.7 mg (±0.7); mean dose of ropinirole 10.9 mg (±7.6 mg). | The median reaction time during the second half of the subtest “vigilance”, test condition “moving bar” of the Test battery for Attention Performances (TAP). | There was no difference in the primary end point reaction time of the TAP subtest vigilance between piribedil and the comparators (996 vs. 954 milliseconds, respectively; p=0.68). | 50% | No safety concerns were identified. |  |
| Rotigotine | Pierantozzi M. Sleep Med (2016)^20^: | PD patients with a disease duration of more than 3 years and Night sleep disturbances (PSQI ≥ 5). | 42 | Patients were randomly assigned to either receive a rotigotine patch (n=21; starting with 2mg/day with a maximum dose of 16mg/day) or a placebo patch (n=21) in a 6- to 10-week active/placebo treatment phase, comprising 4 to 8 weeks of drug titration-to response, followed by 2 weeks of maintenance. Patches were maintained from 18:00 h to awakening, minimizing the possible diurnal impact on motor symptoms. | The effect of rotigotine on sleep macrostructures as assessed by PSG compared to placebo measured by means of two consecutive PSG measures at baseline and at the end of the study | Rotigotine significantly increased sleep efficiency and reduced both wakefulness after sleep onset and sleep latency compared to placebo, while mean change in REM sleep quantity was significantly higher in the rotigotine than placebo group. The improved PSG parameters corresponded to the amelioration of PDSS and PSQI scores together with the improvement of patient morning motor symptoms (as documented by the reduction of UPDRS-III scores). | 70% | No safety concerns were identified. |  |
| **INTERVENTIONS TO TREAT AUTONOMIC DYSFUNCTION IN PD** | | | | | | | | | |
| **INTERVENTIONS TO TREAT ORTHOSTATIC HYPOTENSION IN PD** | | | | | | | | | |
| Droxidopa | Hauser R. A. Journal of Parkinson's disease (2014)^27^: | PD patients (61% male, mean age 72.5 yrs (±7.5)) with documented neurogenic orthostatic hypotension (nOH) (decrease ≥20 mmHg in systolic or ≥10 mmHg in diastolic blood pressure within 3 minutes after going from supine to standing), ≥ 3 on the Orthostatic Hypotension Questionnaire (OHQ) and CGI-S ≥3 (for nOH rated by the study investigator) | 51 | Patients underwent ≤2 weeks of double-blind droxidopa or placebo dosage optimization followed by 8 weeks of maintenance treatment (100–600 mg t.i.d.,). | Change in OHQ composite score from baseline to week 8. | Among 24 droxidopa and 27 placebo recipients, mean OHQ composite-score change at week 8 was −2.2 versus −2.1 (p=0.98) | 76.2% | For dizziness/lightheadedness score, the mean change was −3.1 (±3.4) for droxidopa vs. −1.6 (±3.1) for placebo after week 1 (p=0.24). The mean standing systolic blood-pressure change after week 1 favored droxidopa (+8.4 (±17.4) versus −4.1 (±20.5) mmHg (p=0.04)). Compared with placebo, the droxidopa group exhibited an approximately 50% lower rate of reported falls (p=0.16) and fall-related injuries (post-hoc analysis). | In this preplanned interim efficacy analysis (i.e. study nOH306A), the initial 51 subjects did not demonstrate a significant difference across groups in the trial’s primary efficacy measure which was change in OHQ composite score. Therefore, the original study was stopped for futility based on data from this primary endpoint alone, Subsequently, a corresponding change in the trial’s primary efficacy measure was done while data for subsequent subjects remained blinded (i.e. study nOH306B) ^28^: with resulting analyses of the subsequent 171 enrolled patients of study nOH306. |
| Droxidopa | Hauser R. A. Movement disorders : official journal of the Movement Disorder Society (2015)^28^: | Reported on the subsequent patients enrolled in the above study. PD patients (droxidopa arm: 65% male, mean age 72.5 yrs (±8); placebo arm: 67% male, mean age 71.9 yrs (±7.7)) with documented neurogenic orthostatic hypotension (nOH) | 174 | Patients were randomized 1:1 to receive droxidopa (n=89) or placebo (n=85) (2 weeks titration phase, 8 weeks maintenance phase at each subject’s optimized dosage (100-600 mg TID; mean dosage 436 mg/d). | The primary outcome measure was changed to OHSA (Orthostatic Hypotension Symptom Assessment) item 1 (“dizziness, lightheadedness, feeling faint, or feeling like you might black out”) score change at 1 week. | From baseline to week 1, mean (SD) improvement in OHSA item 1 score was 2.3 (2.95) in the droxidopa group versus 1.3 (3.16) for placebo (difference, -1.0; 95%, confidence interval: -2.0, 0.0; p=0.018). | 80% | AE incidence was similar across groups, but 12.4% of droxidopa and 6.1% of placebo subjects withdrew because of AEs. The most common AEs on droxidopa (vs. placebo) were headache (13.5% vs. 7.3%) and dizziness (10.1% vs. 4.9%). | Due to an interplay between regulatory requirements and the outcomes of other droxidopa trials the primary outcome measure was changed from OHQ composite score from baseline to week 8 to OHSA item 1 (“dizziness, lightheadedness, feeling faint, or feeling like you might black out”) score change at 1 week. |
| **INTERVENTIONS TO TREAT URINARY DYSFUNCTION IN PD** | | | | | | | | | |
| Solifenacin succinat | Zesiewicz T. A. Parkinsonism & related disorders (2015)^38^: | PD patients suffering from overactive bladder (OAB; defined as at least 8 voids per 24 h period and at least daily urinary urgency); aged 40-80 yrs, stable dose of anti- parkinsonian medication 4 weeks prior to study entry, H&Y 1-3, evidence of prostate specific antigen ≤4 (men only) within the last 12 months, and a bladder scan at screening documenting post void residual of 200 ml or less. | 23 | Patients were randomized to receive solifenacin succinate 5-10 mg daily or placebo for 12 weeks followed by an 8-week open label extension. | Change in the mean number of micturitions per 24 h period. | The mean number of micturitions per 24 h period did not significantly improve with the use of solifenacin succinate. T he average number of urinary incontinence episodes per 24 h period decreased significantly in the solifenacin group (1.48 ± 2.56 to 0.30 ± 0.31) compared to placebo (1.78 ± 1.27 to 1.61 ± 1.40, p=0.01). | 90% | AEs included constipation and xerostomia, which resolved after treatment was discontinued. |  |
| **INTERVENTIONS TO TREAT ERECTILE DYSFUNCTION IN PD** | | | | | | | | | |
| Sildenafil | Bernard Bryan A. Movement Disorders Clinical Practice (2017)^10^: | PD HY 1-3 in the ON state, (mean age: 60 yrs (±7.7); mean disease duration: 7.8 yrs (±5.9)), with erectile dysfunction (ED; an inability to achieve an erection sufficient for intercourse more than 50% of attempts during the preceding 3 months). | 20 | Patients were randomized using a random-length permuted block design to either 50 mg of sildenafil or placebo. After 2 weeks of study medication the dose was increased to 100 mg or matching placebo for the second 2-week period. If side effects occurred but were considered mild the dose was reduced to 25 mg for the rest of the study. | Erectile Function domain of the International Index of Erectile Function (IIEF) | There was a significant effect of sildenafil on sexual functioning as measured by the IIEF-EF domain (p<0.0001; mean for sildenafil: 23.2+/-7.0; mean for placebo: 12.3 +/- 7.5). | 81.6% | There were no safety concerns in this study. |  |
| **INTERVENTIONS TO TREAT SIALORRHEA IN PD** | | | | | | | | | |
| Botulinum toxin type B (BoNT-B) | Chinnapongse R. Movement disorders : official journal of the Movement Disorder Society (2012)^13^: |  | 49 | Patients were randomized to receive one of three dosages of BoNT-B (1.500U, n=13 - 2.500U, n=10 - 3.500U, n=12) or placebo (n=12). | Safety/tolerability | Overall BoNT-B appears safe and all three BoNT-B dosages significantly improved most of the efficacy outcomes. | 81% | No new safety concerns identified. |  |
| **INTERVENTIONS TO TREAT CONSTIPATION IN PD** | | | | | | | | | |
| Lubiprostone | Ondo W. G. Neurology (2012)^12^: | PD | 52 | Patients were randomized to receive either lubiprostone (n=25; a locally acting chloride channel activator that enhances chloride-rich intestinal fluid secretion without altering sodium and potassium concentrations in the serum) or placebo (n=27). | No clear defined primary outcome measure. | There were significant increased stools per day by diary in lubiprostone versus placebo after 4 weeks (lubiprostone: from 0.75±0.80 to 0.97±0.88, placebo: from 0.84±0.76 to 0.83±0.76; p=0.001), a significant improved visual analog scale score in lubiprostone versus placebo (p=0.001) and a significant improved constipation questionnaire in lubiprostone versus placebo (p=0.033). “Much” or “very much” improved constipation on the CGIC was observed in 64% of lubiprostone treated patients versus 19% of the placebo treated patients after 4 weeks. | 71% | There were no safety concerns in this study. |  |
| Probiotics and prebiotic fiber | Barichella M. Neurology (2016)^40^: | PD patients meeting Rome III criteria for functional constipation. | 120 | Patients were randomized to receive either fermented milk, containing multiple probiotic strains and prebiotic fiber, or placebo (fermented, fiber-free milk), once daily at breakfast for four weeks. | Number of complete bowel movements after four weeks assessed with the use of a stool diary. | The number of complete bowel movements increased with consumption of fermented milk with probiotics and prebiotic fiber (mean 1.2, 95% confidence interval [CI] 0.8–1.6) compared to the placebo group (0.1, 95% CI −0.4% to 0.6%; p = 0.002). | 89.5% | There were no significant differences in AEs between the treatment and the placebo group. Two patients of the active arm reported bloating and abdominal distension. |  |
| Abdominal massages | McClurg D. Parkinson's disease (2016)^39^: | PD patients with self-reported constipation. | 32 | Patients were randomized to receive either 6 weeks of daily abdominal massages and lifestyle advice (n=16) or lifestyle advice only (n=16). | Effect on the bowel dysfunction questionnaires compared at baseline, week 6 of treatment and 4 weeks after end-of-treatment. | There was no significant group difference between the intervention and the placebo groups (p=0.477), there were, however, improved bowel dysfunction questionnaire results at 6 and 10 weeks in both groups with no between-group differences. | 52.5% | AEs or side effects are not mentioned in this study. |  |
| **INTERVENTIONS TO TREAT OTHER NON-MOTOR SYMPTOMS IN PD** | | | | | | | | | |
| **INTERVENTIONS TO TREAT FATIGUE IN PD** | | | | | | | | | |
| Rasagiline | Lim T. T. Movement disorders : official journal of the Movement Disorder Society (2015)^24^: | PD patients naïve to rasagiline / selegiline. | 30 | Patients were randomized 1:1 to receive rasagiline (1mg) or placebo. | Change in severity of fatigue scored using the Modified Fatigue Impact Scale (MFIS) between baseline and 12 weeks after treatment. | There was a significant improvement in the MFIS score of the active group compared to the placebo group from baseline to week 12 (12 vs. 8 points, p=0.003). | 76.3% | There were no significant AEs in either group. |  |
| Acupuncture | Kluger B. M. Movement disorders : official journal of the Movement Disorder Society (2016)^25^: | PD patients with moderate-to-high fatigue. | 94 | Patients were randomized to receive real or sham acupuncture twice a week for 6 weeks, followed by a follow-up over additional 6 weeks. | Difference in MFIS between the two arms. | There were no differences in MFIS scores between the intervention and the sham acupuncture arm at six weeks (p=0.44). | 71.1% | There were no differences regarding the occurrence of side effects between the two groups, and there were no serious AEs were observed. |  |
| **INTERVENTIONS TO TREAT PAIN IN PD** | | | | | | | | | |
| Oxycodone-naloxone prolonged release | Trenkwalder C. The Lancet Neurology (2015)^26^: | PD (H&Y II-IV) and chronic, severe pain (average 24-h pain score ≥ 6 and severe pain in at least one subsection of the Chaudhuri and Schapira pain classification system). | 202 | Patients were randomly assigned to receive oxycodone-naloxone prolonged release (n=93; mean dose 18.8 mg ± 8.4) or matching placebo (n=109; mean dose 23.5 mg ± 8.9). 67% in the active treatment group and 71% in the placebo group completed the study. | Average 24-h pain score at week 16. | The reduction in the average 24-h pain score at 16 weeks did not differ significantly between groups (least square mean 5.0 (95% CI 4.5 - 5.5) in the OXN PR group vs. 5.6 (5.1 - 6.0) in the placebo group (difference –0.6, 95% CI –1.3 - 0.0; p=0.058). Pain significantly improved in patients with severe musculoskeletal PD pain and with severe nocturnal pain on active vs. placebo treatment. | 83.3% | Treatment-related nausea was more common with OXN PR than with placebo (17% vs. 9%), as was treatment-related constipation (17% vs. 6%). There were no safety concerns in this study. |  |
| Rotigotine | Rascol O. Journal of clinical pharmacology (2016)^30^: | Advanced PD (defined by use of levodopa ≥ 200 mg/day) and at least moderate PD-associated chronic pain (≥3 months, ≥4 points on 11-point Likert pain scale). | 68 | Patients were randomized to receive rotigotine (optimal/maximum dose 4-16 mg/24h; mean dose 14.7 (±5.1) mg/24 h) or placebo and maintained for 12 weeks | Change in pain severity (Likert pain scale) from baseline to end of maintenance. | At the end of the 12-week maintenance period, a numerical improvement in the average pain severity experienced in the last 7 days (Likert pain scale) was observed in favor of rotigotine (least-squares [LS] mean [95%CI] treatment difference, –0.76 [–1.87 to 0.34]; p=0.172). | 82.6% | There were no safety concerns in this study. |  |

**References**

See main text
